# Mass Spectrometry Imaging Reveals the Distribution of a GABRG2 Targeting Antisense Oligonucleotide and Its Functional Effect in Rat Brain

**DOI:** 10.1021/acschemneuro.5c00524

**Published:** 2025-10-01

**Authors:** Laura van der Vloet, Ronny Mohren, Christophe Bouillod, Georges Da Violante, Emre M. Isin, Ron M. A. Heeren, Pierre Barbier Saint Hilaire, Michiel Vandenbosch

**Affiliations:** † The Maastricht MultiModal Molecular Imaging (M4I) institute, Division of Imaging Mass Spectrometry (IMS), 5211 Maastricht University, Maastricht 6229 ER, The Netherlands; ‡ Institut de Recherche et Développement SERVIER Paris-Saclay, 22 route 128, Gif-sur-Yvette 91190, France

**Keywords:** antisense oligonucleotides, matrix-assisted laser desorption/ionization mass spectrometry imaging, multiomics, central nervous system, γ-aminobutyric acid A receptor γ2 subunit, neurotransmitters

## Abstract

In recent years, the development of antisense oligonucleotides (ASOs) has gained wide interest as therapeutic agents for their potential in treating neurodegenerative diseases. ASOs are chemically modified oligonucleotides that are designed to bind complementary regions of RNA or DNA and, thereby, modulate the expression of the corresponding protein. Here, we present a multiomics approach to investigate the spatial distribution and biological effect of an ASO designed to target the mRNA that translates for γ-aminobutyric acid A receptor γ2 subunit (GABRG2), which is abundantly expressed within the brain. In this study, a rat model was used to develop a multiomics mass spectrometry (imaging) approach to map ASO distribution in brain and kidney, followed by in-depth analysis of the lipidome, proteome, and metabolome. The ASOs’ phosphorothioate-modified backbone was visualized using an optimized matrix-assisted laser desorption/ionization mass spectrometry imaging (MALDI-MSI) protocol, which included the introduction of an organic washing step prior to MALDI-MSI acquisition and an optimized acquisition method. On consecutive tissue sections, reactive matrix FMP10 was applied to enable the visualization of neurotransmitters, which revealed significant alterations for multiple neurotransmitters. Lastly, on the same slide, the ASOs’ effect on the lipidome and proteome of the brain was further analyzed. Proteins corresponding to synaptic activity and plasticity were mainly affected by the ASO. This spatial omics approach provides insight into the comprehensive molecular landscape of ASO-mediated interventions and their promise as treatments for neurological disorders.

## Graphical abstract

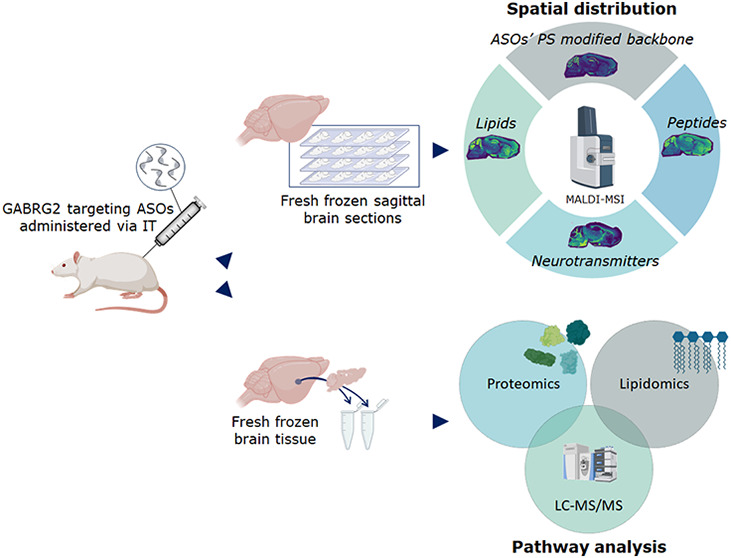

## Introduction

In the dynamic field of drug discovery and development, it is crucial to have a comprehensive understanding of a drug’s absorption, distribution, metabolism, excretion, and toxicity, as well as on its effects on biological pathways. As part of the recent evolution of precision medicine approaches in drug discovery and development, antisense oligonucleotides (ASOs) have shown promise to revolutionize the treatment of genetically defined rare disease and central nervous system (CNS) disorders that in most cases are severely debilitating and still lacking an adequate treatment.^1^ ASOs are short, synthetic, single-stranded oligodeoxynucleotides that can alter RNA, resulting in reduced, restored, or modified protein expression by selectively binding to the RNA through complementary base pairing.^2^ ASOs have been proven to be a promising drug candidate for the treatment of CNS disorders when they are delivered directly into the CNS. However, the spatial distribution and the pharmacokinetics (PK) and pharmacodynamics (PD) of intrathecal (IT)-dosed ASOs remain poorly understood. Many molecules can exchange between the cerebrospinal fluid (CSF) and the CNS interstitial fluid (ISF); however, the exact physiological processes connecting CSF and ISF, and their effect on underlying biological mechanism, are still extensively being investigated.^3^ Despite their proven therapeutic value, some ASOs are associated with toxic effects that restrict their use. Systemically and IT-administered ASOs distribute to various tissues and then accumulate particularly in the kidney and liver.^4^ An effective and fast screening tool to determine the distribution of ASOs and their biochemical effects is still lacking.

The ASO used in this study targets the mRNA that translates into a subunit of the γ-aminobutyric acid A receptor (GABA_A_R). This is a heteropentameric complex that can be composed of combinations of 19 different polypeptide subunits, including six α (α) 1–6, three β (β) 1–3, three γ (γ) 1–3, three rho (ρ) 1–3, or one delta (δ), epsilon (ε), pi (π), or theta (θ), resulting in multiple receptor isoforms. Each specific isoform exhibits unique pharmacological and physiological properties. The majority of GABA_A_Rs are composed of two α subunits, two β subunits, and one γ subunit, specifically consisting of the γ2β2α1β2α1.^5^ GABA_A_Rs are ligand-gated ion channels that are abundant in the CNS, functioning as primary mediators of fast inhibitory neurotransmission in the brain once GABA binds between the α and β subunits. Activation of the GABA_A_R results in opening the chloride channel, followed by an increase of the chloride influx into the postsynaptic neuron.^6^ GABA is synthesized in the cytoplasm of presynaptic neurons from the precursor glutamate. In contrast, glutamate causes depolarization of the postsynaptic neuron, thus generating excitatory postsynaptic potentials, most likely generating an action potential.^7^ GABA-glutamate imbalance and GABA_A_R dysfunction are associated with multiple neurological and neurodevelopment disorders, including epilepsy, schizophrenia, autism, insomnia, and anxiety disorders.^6,8,9^ Drugs that modulate GABA_A_R activation are found to be effective as anticonvulsants, anxiolytics, antidepressants, and general anesthetics. These therapeutics can bind to multiple sites on the receptors and, therefore, act synergistically to potentiate GABA_A_R activation. Among the binding sites, the benzodiazepine site is the best characterized so far. Benzodiazepines bind to an α subunit and the γ2 subunit, and represent the most successful psychotropic drug class, which is used to treat insomnia.^8^ However, given the high-affinity binding site at the α-γ subunit interface, severe side effects can occur following benzodiazepine administration that is caused by their nonselective targeting of GABA_A_Rs.^10^ In this work, the ASO specifically binds and degrades the RNA coding for the translation of the GABA_A_R γ2 subunit (GABRG2). GABRG2 is crucial for normal channel function and for postsynaptic clustering during synaptogenesis. The loss of postsynaptic GABA_A_Rs reduces channel function. Additionally, GABRG2 is involved in stabilizing and trafficking of GABA_A_R to and from the cell surface.

The multiomics approach presented in this study provides an in-depth analysis of an ASO candidate that targets the mRNA that translates into GABRG2 in brain tissue. For the first time, the ASO distribution was visualized in brain tissue using matrix-assisted laser desorption/ionization mass spectrometry imaging (MALDI-MSI), followed by analyzing the functional effects, including the metabolome, proteome, and lipidome. To our knowledge, only three other studies demonstrated ASO distribution utilizing MALDI-MSI, mainly focusing on kidney tissue.^4,11,12^ Here, we present an optimized MALDI-MSI method to enable the visualization of ASOs in brain tissue, which is known to contain lower ASO concentrations compared to kidney tissue. ASO visualization via MALDI-MSI overcomes multiple limitations of immunohistochemistry, *in situ* hybridization (ISH), single photon emission computed tomography/computed tomography (SPECT/CT) live imaging, or cryo-fluorescence tomography, which have been used in studies to visualize ASOs in biological tissues.^3,13^ MALDI-MSI requires no prior labeling, enabling the detection of the initial therapeutic drug and its metabolites. Thereby, it provides the possibility to analyze multiple omics on a single tissue section, followed by a histological staining. Here, MALDI-MSI proved to be an effective strategy to study different molecules, including the ASOs’ modified phosphorothioate (PS) backbone, lipids, peptides, and neurotransmitters. In this study, we present a comprehensive overview of the ASOs’ biological effect, inhibiting the GABRG2. To improve the understanding of the ASOs’ biological effect on the lipidome and proteome, we combined MALDI-MSI with liquid chromatography tandem mass spectrometry (LC-MS/MS) analysis. Combining these methodologies holds significant importance in drug discovery and development research, which mainly focuses on the in-depth understanding of the spatial and molecular dynamics within biological tissue samples. This spatial omics approach offers insight into the comprehensive molecular landscape of ASO-mediated interventions and their potential as therapeutic treatments for neurological disorders.

## Results and Discussion

### Matrix-Assisted Laser Desorption/Ionization MSI of the Phosphorothioate-Modified ASO Backbone in Kidney and Brain Tissue

In this study, we aimed to visualize the intact ASO and its metabolites in kidney and brain tissue using MALDI-MSI. By utilizing MALDI-MSI, we should be able to distinguish the initial ASO with its metabolites as it does not require any prior labeling of the ASO. The ASO used in this study had a molecular weight of 6559.58 Da, containing several chemical modifications to enhance the stability and increase the half-life of the ASO, including PS modifications on the backbone and 2′-O-methoxyethyl (2′-MOE) modification on the sugar group. Supporting Information Figure S1 presents an example of an ASO structure containing the aforementioned modifications. Studies have already described optimized sample preparation methods that are required prior MALDI-MSI analysis to detect and visualize ASOs.^11,12^ This included the introduction of polar washing steps, such as ethanol, acetone, and Carnoy’s solution, which were used to remove lipids from the biological samples, and thus reduce ion suppression and enhance sensitivity.^11,12,14^ However, using MALDI-MSI, we were unable to detect the initial ASO or any metabolites. Therefore, we focused on visualizing a fragment originating from the ASO backbone that consisted of a PS modification to localize ASO fragments in kidney and brain tissue.^4^ The signal detected from the PS-modified backbone can orientate either from the initial ASO molecule or from any potential metabolites. However, ASO metabolism is limited in the brain, suggesting that the detected signal is mainly linked to the initial ASO molecule. Since ASOs cannot cross the blood–brain barrier, the concentration of the ASO is also lower in brain tissue compared to the kidney in which ASOs are known to accumulate.^1,3,4^ Prior to MALDI-MSI analysis, we introduced multiple washing solutions and time points to optimize the sample preparation to enhance the sensitivity of the PS-modified backbone of the ASO (Supporting Information Figures S2 and S3). Finally, washing the tissues for 60 s with dichloromethane (DCM) showed significantly the highest intensity of the PS-modified backbone in brain tissue. Besides an optimized sample preparation workflow, the TimsIn pressure of the Timstof was decreased to 1.8 mbar to increase the sensitivity of the ASO backbone fragment.

The PS-modified backbone (*m*/*z* 94.94) was mainly observed in the renal cortex of rats that were sacrificed 8 and 15 days post ASO administration (Figure 1A). The levels of the PS-modified backbone were significantly higher in the renal cortex of rats sacrificed 8 days post administration compared to rats sacrificed 15 days post administration. The PS-modified backbone of ASO was not detected in the control. In brain tissue, the ASO backbone fragment was only visible in rats sacrificed 8 days post ASO administration. The ASO backbone fragment in brain tissue was only detectable between the cortex and midbrain and around the edges of the cerebellum. We hypothesized that the ASOs that were not bound to their target RNA in the brain were visualized. Depending on the ASOs’ chemistry and modifications, the ionization of the ASO can be influenced.^4,11,12^ When ASOs are hybridized with its target RNA via Watson–Crick base pairing, the resulting duplex has a different conformation, stronger intermolecular interactions, and potential of shielding the negatively charged backbone.^15^ This can all lead to a lower desorption and ionization efficiency when utilizing MALDI-MSI. The overall abundance of the ASO fragment was also lower in brain tissue compared to kidney or liver tissue. Depending on the ASOs chemistry and final concentration in brain tissue, it is possible that the abundance of the ASO is too low, resulting in the limit of detection value of the ASO being too high.

**Figure 1:**
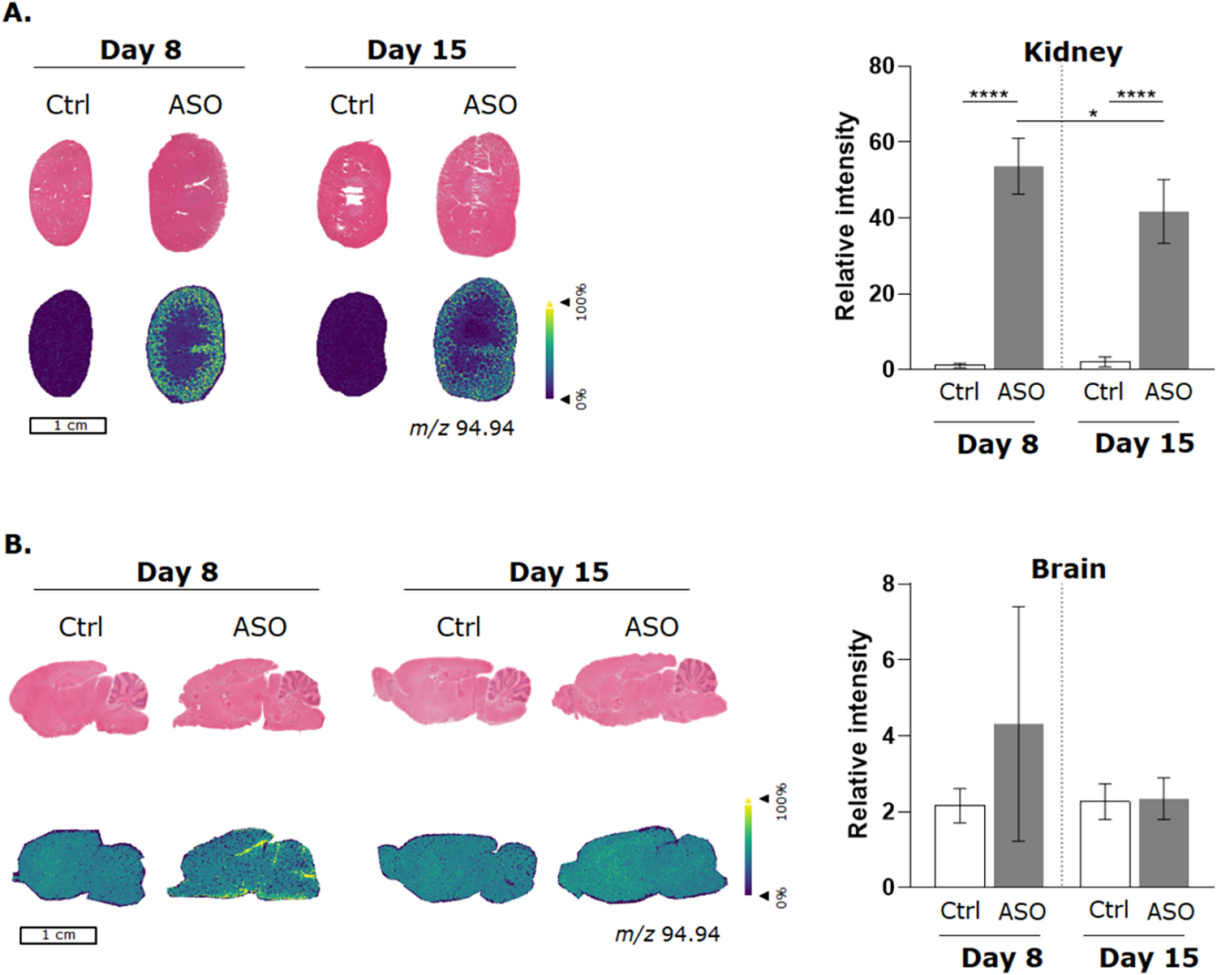
Spatial distribution of the ASOs’ PS-modified backbone fragment. (A) The distribution of the PS-modified backbone of the ASO in the kidney was visualized with MALDI-MSI, in which yellow/green indicates higher levels of the ASO fragment compared to purple regions within the tissue. The relative intensity is presented in bar plots. The error bar represents the standard deviation between the biological replicates (*N* = 3 control and *N* = 2 ASO dosed). (B) The ASOs’ PS-modified backbone distribution in brain tissue and their corresponding H&E staining. A one-way ANOVA statistical test was performed to determine the significance between the groups. *p < 0.05, and **** *p* < 0.0001.

A human brain transcriptome analysis showed that among the high expression genes of GABA_A_R subunits, the α1, β2, and γ2 contributed up to 48% of the global gene expression for GABA_A_Rs across the brain.^16^ GABRG2 is highly expressed in the cerebral cortex, specifically in the dentate gyrus of the hippocampal formation.^16,17^ To assess whether the GABRG targeting ASO did bind in these specific brain regions, a targeted approach should be utilized such as classical ISH or MALDI-ISH-MSI.^18^

### GABRG2 Inhibition Reduces GABA, Dopamine, and α-Tocopherol Levels in the Brain

By targeting *GABRG2*, the γ2 subunit protein translation was inhibited. The metabolome, specifically neurotransmitter levels and distribution, was evaluated in the brain. To enable the detection and quantification of neurotransmitters utilizing MALDI-MSI, internal standards (GABA-d6 (*m*/*z* 377.21) and DA-d4 (*m*/*z* 425.21)), followed by a reactive matrix, were applied prior to analysis. Supporting Information Figure S4. A presents the reaction between the reactive matrix FMP10 and GABA, which forms the FMP10-GABA complex (*m*/*z* 371.17). In total, 17 neurotransmitters could be visualized in sagittal rat brain sections. An H&E staining was performed on a consecutive slide to annotate the different brain regions (Supporting Information Figure S4C) to evaluate neurotransmitter levels per region. High-mass-resolution Fourier transform ion cyclotron resonance (FT-ICR-)­MSI was performed to confirm neurotransmitter IDs (Supporting Information Table S1). A principal component analysis (PCA) was performed on those 17 neurotransmitters, which separated the control from the 8 days post administration ASO brains (Supporting Information Figure S4B). This provided the first evidence that neurotransmitters are affected by inhibiting GABRG2. The relative intensities of all detected neurotransmitters per brain region were visualized in bar plots (Supporting Information Figures S5 and S6). In most brain regions, a trend of reduction in GABA levels was observed in GABRG2-inhibited tissues that were sacrificed 8 days post ASO administration. The highest levels of GABA were observed in the basal forebrain and hypothalamus (Supporting Information Figure S5). GABA was only significantly decreased in the midbrain when inhibiting GABRG2 (8 days post sacrifice) (Figure 2B). GABA levels were no longer observed to be altered in brain tissue 15 days post administration (Supporting Information Figure S5). GABA concentrations are generally higher in gray matter compared to white matter, since gray matter contain neuronal cell bodies and synapses.^19,20^ In a healthy state, high levels of GABA are found in the cerebellum, basal ganglia and thalamus.^21^ GABAergic neurons are located in multiple brain regions, including the hippocampus, thalamus, basal ganglia, hypothalamus, and brainstem.^16,17^ GABA functions as the primary inhibitory neurotransmitter that is synthesized from glutamate. Glutamate is the primary excitatory neurotransmitter in the brain that leads to a reduction of neuronal excitability. This is caused by neuronal hyperpolarization and reducing neurotransmitter release.^7,22^ Mechanisms maintaining the balance between glutamate-mediated synaptic excitation and GABA-mediated synaptic inhibition are crucial for normal physiological brain functions.^23^

**Figure 2:**
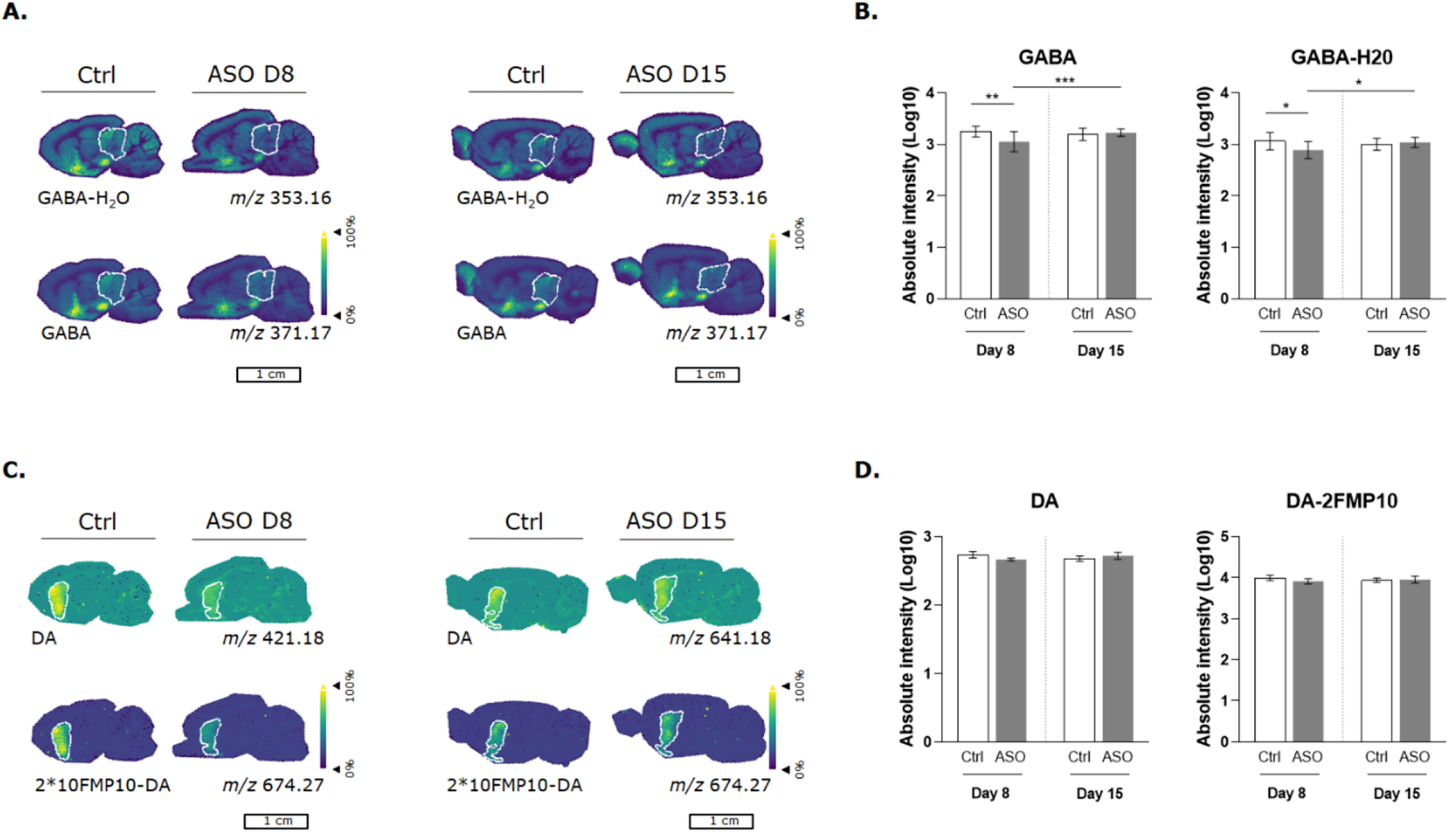
GABA and DA distribution in control and ASO dosed sagittal rat brain sections 8 and 15 days post administration. (A) Ion images correspond to GABA-H_2_O (*m*/*z* 353.16) and GABA (*m*/*z* 371.17) in sagittal rat brain sections. (B) Relative intensities (Log10) normalized to their corresponding internal values of GABA-H_2_O and GABA in the midbrain are presented in bar plots. (C) Ion images correspond to DA (*m*/*z* 421.18) and 2*FMP10-DA (*m*/*z* 674.27) in brain sections. (D) Relative intensities (Log10) normalized to their corresponding internal standard of DA and 2*FMP10-DA in the striatum are presented in bar plots. Ion images are normalized to their corresponding internal standard: GABA-d6 (*m*/*z* 377.21) and DA-d4 (*m*/*z* 425.21). The error bar represents the standard deviation between the biological replicates (*N* = 3 control and *N* = 2 ASO dosed). Significance was determined by performing a 2-way ANOVA. Error bars present the standard deviation. *p < 0.05, **p < 0.01 and *** *p* < 0.001.

Besides GABA that was affected by inhibiting GABRG2, a reduction trend of dopamine was observed 8 days post ASO administration in the striatum (Figure 2C,D). Dopamine was primarily present in the striatum of the brain, which is a crucial regulator of striatal neuronal output.^24^ Overall, dopamine was not significantly altered in a specific brain region (Supporting Information Figure S5). The magnitude of dopamine is influenced by other striatal present neurotransmitters, including GABA and glutamate.^24^ However, we did not observe any correlation between GABA and dopamine in the striatum, as no significant alteration in GABA levels was observed in the striatum.

Interestingly, we found α-tocopherol (the most biologically active form of vitamin E) to be significantly reduced in the basal forebrain, brainstem, hypothalamus, and midbrain (Supporting Information Figure S6). α-Tocopherol is an essential supplement that is essential for maintaining healthy brain function and functions as a major lipid-soluble antioxidant. The downregulation of α-Tocopherol suggests reduced antioxidant protection, making the brain more susceptible to oxidative stress and neurodegeneration.^25^

### Inhibiting GABRG2 Affects Synaptic Regulation and Plasticity

By inhibiting the translation of GABRG2 by targeting the corresponding mRNA, we investigated the effect of this inhibition further on the brain proteome. Untargeted spatial proteomics was combined with LC-MS/MS proteomics analysis to evaluate protein alterations in specific brain regions. A PCA was performed on the LC-MS/MS proteomics data set to analyze whether the control group could be separated from the ASO-treated groups. Figure 3A presents the PCA plot, showing a separation of the control group from the 8 days post ASO administration group. A 95% confidence ellipse is added to each group. However, the control group could not be separated from the ASO-treated group 15 days post administration (Supporting Information Figure S8A). All proteins with a false discovery rate confidence lower than 1% are plotted into a volcano plot (Figure 3B). Significantly altered proteins are presented in green, in which 135 proteins were upregulated in the ASO-treated brain and 60 proteins were downregulated (*p* < 0.05 and Log_2_ Fold Change >1.04). Interestingly, GABRG2 was not found in the data set. Some proteins are highlighted that were upregulated in the ASO-treated group, including GABA_A_ receptor α1 subunit (GABRA1), glutamate ionotropic receptor NMDA type subunit 2B (GRIN2B), and glutamate ionotropic receptor AMPA type subunit 2 (GRIA2). These proteins are subunits of the GABA_A_R, NMDA, and AMPA receptors, respectively. As previously discussed, a balance in excitatory and inhibitory (E/I) neurotransmitters is crucial for normal brain functioning. E/I imbalance contributes to the pathobiology of neurological disorders.^7,23^ The amount of GABA_A_Rs in the membrane surface and at synaptic sites is an important determinant of the inhibitory strength of synapses. The activation of ionotropic glutamate receptors during plasticity and in pathology can result in the reduction of inhibitory synapse strength and GABA_A_R function. The rapid movement of neurotransmitter receptors within synapses contributes to regulating synaptic strength as well.^26^ Neuroligin-neurexin complexes are heterophilic adhesion systems that are broadly expressed in the CNS and are common building blocks of glutamateric and GABAeric synapses. They are required for normal glutamateric and GABAeric transmission, and crucial for the organization of GABAeric synapses.^27^ Neuroligin (NLGN2) was upregulated in the ASO-treated group 8 days post administration. Proteins like GAP43 are a crucial component of the axon and presynaptic terminal that is associated with nerve growth and presynaptic membrane changes,^28^ which were also found to be increased in the ASO-treated group.

**Figure 3:**
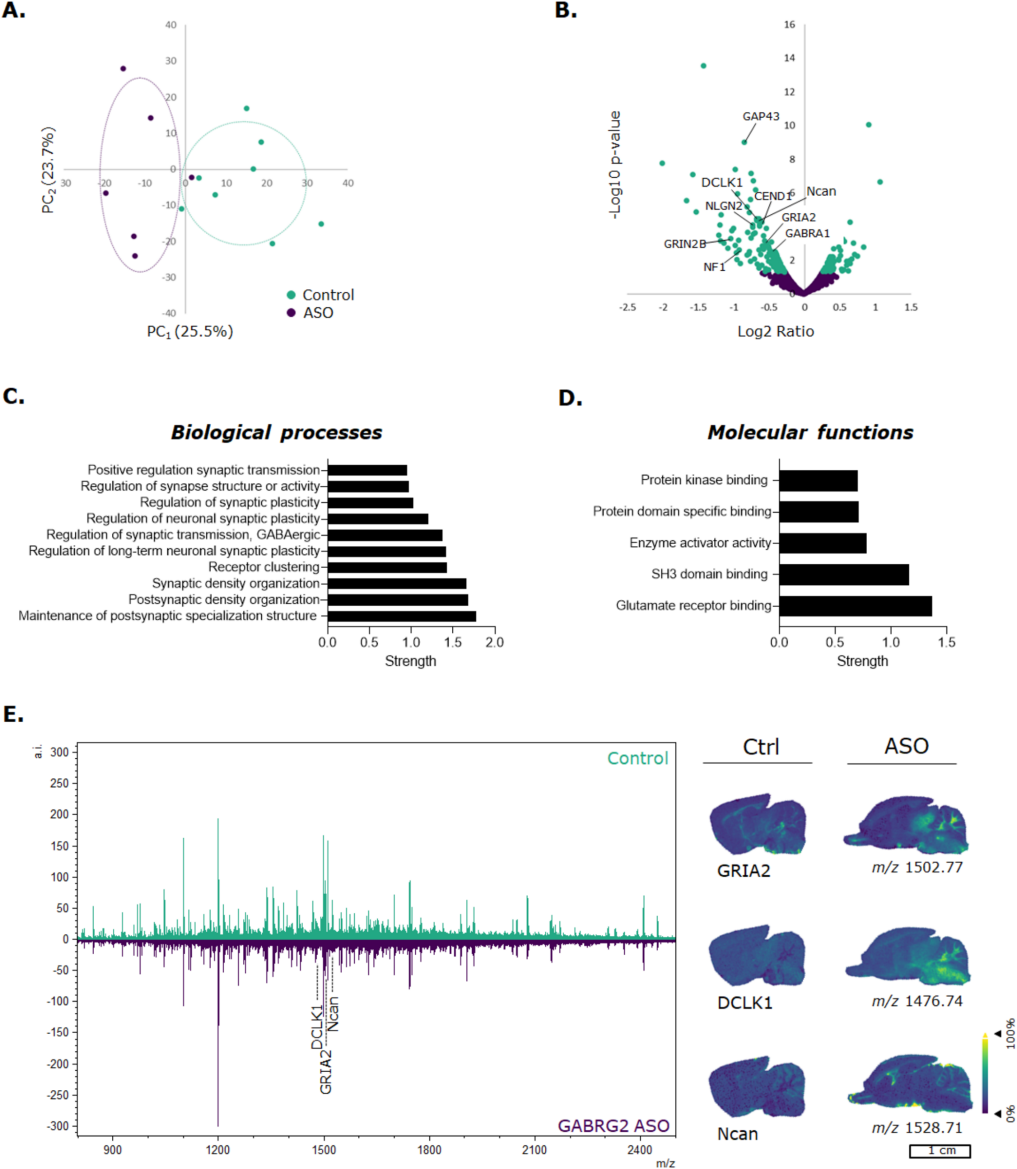
(Spatial) proteomic pathway analysis of brain tissue 8 days post ASO administration. (A) PCA of identified proteins from the LC-MS/MS data set showed a false discovery rate lower than 1%. In green, the control tissues are presented, and in purple, the ASO dosed (8 days after administration) are presented. A 95% convenience ellipse is presented per experimental group (*N* = 3 control and 2 ASO dosed). (B) A volcano of identified proteins from the LC-MS/MS data set with a false discovery rate lower than 1%. In green, the significantly (*p* < 0.05) altered proteins are presented. The left side of the volcano plot presents proteins that are upregulated in ASO dosed (8 days post administration) tissues, as where the right side presents proteins that are downregulated in ASO dosed tissues. (C, D) String protein pathways (the top 10 GO terms related to biological processes and molecular functions) from significantly altered proteins in ASO dosed brain sections. (E) Spatial distribution of significantly altered peptides between control and ASO dosed tissue sections. An average spectrum of all control tissues (*N* = 3) in green and ASO dosed tissues (*N* = 2) in purple are presented. Ion images are root-mean-square normalized.

The significantly altered proteins (Supporting Information Tables S2 and S3) were then used to perform pathway analysis. Figure 3C,D presents the top 10 biological processes that are affected by the ASO, and their corresponding molecular functions. Overall, synaptic regulation and plasticity were mainly affected in the ASO-treated group 8 days post administration. Protein pathway analysis was also performed in Reactome, confirming that the neuronal system was affected by ASO administration, especially highlighting neurotransmitter receptors that were affected (Supporting Information Figure S7). After 15 days post administration, neuronal cell development and orientation were the main pathways affected (Supporting Information Figure S8C,D). This in-depth proteomics analysis revealed that our ASO influences not only the GABA_A_Rs, but also glutamate receptors (NMDA and AMPA). These receptors are important for strong synapse potentials and are closely related to each other. GABRG2 is essential for the accumulation of cell surface GABA_A_Rs at the postsynaptic site. Acute inhibition of GABRG2 in cultured hippocampal neurons caused altered GABA_A_R clustering and reduced GABAergic transmission.^29,30^

Lastly, spatial proteomics was performed to assess the spatial distribution of peptides that corresponded to the proteins that were significantly altered after ASO administration. Since MALDI-MSI is less sensitive than LC-MS/MS, a lower amount of peptides could be detected. Three peptides could be identified that correspond to significantly altered proteins that were found in the LC-MS/MS data set. The spatial distribution of GRIA2, doublecortin-like kinase 1 (DCLK1), and neurocan are presented in control and ASO-treated brain tissue (Figure 3E). GRIA is highly expressed in Purkinje cells that are located in the cerebellum.^31^ An increase in the level of GRIA2 was mainly observed in the cerebellum. The spatial proteomics data confirm the upregulation of these proteins in brain tissue that also were found in the LC-MS/MS data. Taken together, these data suggest that inhibiting GABRG2 significantly alters synaptic regulation and affects other neurotransmitter receptors that are essential for E/I balance.

### Slight Alterations in Lipid Composition as a Result of Inhibiting GABRG2

The proteome analysis revealed significant alterations in subunits of important membrane proteins that are involved in neurotransmitter regulation. The lipidome, an abundant compartment of cell membranes, was further analyzed utilizing untargeted LC-MS/MS lipidomics analysis that was complemented with MALDI-MSI. It is known that cholesterol in particular has an important regulatory function in GABA_A_R functionality, affecting its responsiveness to various modulators. Astrocytes are the brain’s primary cholesterol producer, secreting cholesterol that are essential for functional integrity of proteins within lipid compartments. Cholesterol increases the GABA_A_R’s channel’s association with lipid rafts in neurons. The γ2 subunit directs the localization of GABA_A_Rs within lipid rafts and within the synapse.^32^ In this study, lipids were measured in both positive and negative ionization modes, in which we could detect 815 different lipids. From those lipids, glycerophospholipids were the most abundant lipid group that was detected in the brain (Supporting Information Figure S9). Neurons require a large amount of membrane lipids to cover their axons, dendrites, and synapses.^33^ Lipids are heavily involved in the regulation of synapse development and plasticity, and presynaptic vesicle release.^34^ Defects in lipid metabolism can lead to structural and functional CNS diseases.^33^ Synapses have lipid composition that distinct from the rest of the cell membrane, suggesting that neurotransmitter receptors (e.g., GABA_A_, NMDA, and AMPA), their scaffolding, and adaptor proteins require specific lipid habitats for normal functioning.^35^

Lipid levels were evaluated between control and 8 days post ASO administration (Figure 4A,B), and control and 15 days post ASO administration (Figure 4C,D). After 8 days of ASO administration, only 16 lipids were significantly altered (Supporting Information Table S4), of which most lipids were upregulated in the ASO-treated group (Figure 4A). MALDI-MSI was used to visualize lipids and assess their spatial distribution (Figure 4B,D). After 15 days post ASO administration, a total of 46 lipids were significantly altered (Supporting Information Table S5), of which most were also upregulated in the ASO-treated group (Figure 4C). In both ASO groups, we observed that plasmalogens were significantly altered. Plasmalogens make up a class of membrane glycerophospholipids that contain a fatty alcohol with a vinyl-ether bond at the *sn-*1 position and are enriched in polyunsaturated fatty acids at the *sn-*2 position of the glycerol backbone. Plasmalogen lipids alter membrane properties of glycerophospholipids, providing unique structural attributes, facilitating signaling processes, and protecting membrane lipids from oxidation.^36^ Glycerophospholipids were mainly affected in the brain by inhibiting GABRG2 after 8 and 15 days of ASO administration. Glycerophospholipids provide neural membranes with stability, fluidity, and permeability.^37^ Neuronal membranes contain besides lipids also transmembrane and peripheral proteins of various shapes, molecular masses, and charges. The binding of these proteins to glycerophospholipids is essential for vertical positioning and tight integration in the lipid bilayer, but also for the optimal functioning of the receptors, ion channels, and membrane-bound enzymes.^38^ Normal functioning of the lipidome is thus crucial for optimal neurotransmitter regulation and E/I balance. An imbalance can cause functional CNS diseases. These data suggest that targeting GABRG2 slightly affects the lipidome. Using a multiomics approach covering the metabolome, proteome, and lipidome is therefore crucial to investigate the biological effect of the therapeutic ASO, but also to identify possible unwanted site effects. Since ASOs are emerging therapeutic strategies to, among others, treat CNS-related disorders and diseases, it is crucial to have in-depth knowledge on the PK/PD. Multiomics (imaging) approaches as presented here can be a valuable tool to unravel disease models, followed by the assessment of a therapeutic drug candidate’s distribution, its effect in tissue, and possible toxic off-target effects.

**Figure 4:**
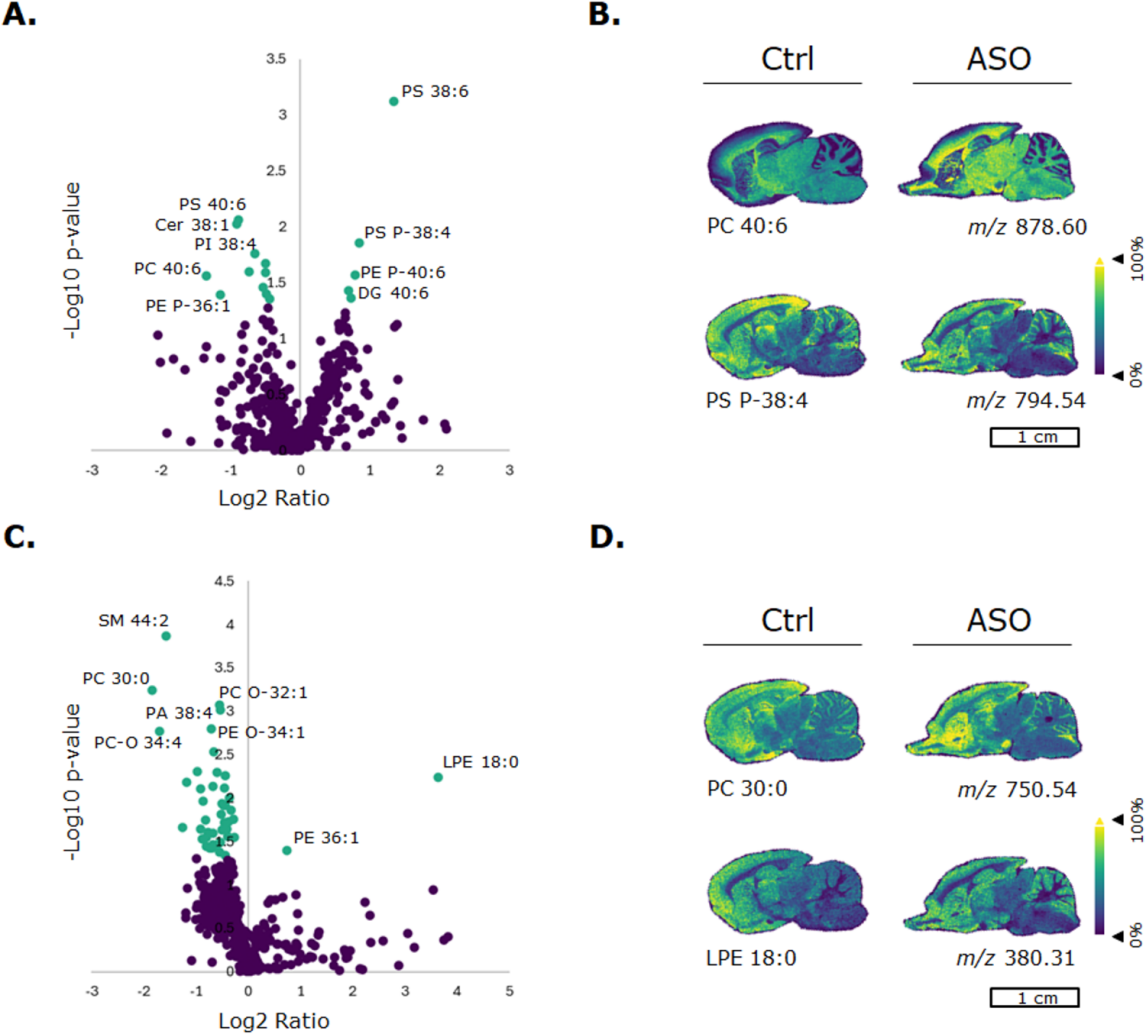
(Spatial) lipidomics analysis of GABRG2-inhibited brain, 8 and 15 days post ASO administration. (A, B) Control vs ASO 8 days post administration: Volcano plot of identified lipids (LC-MS/MS data set) classified with 3- and 4-stars in Lipostar. Positive and negative ionization mode measurements are combined. A lipid is significantly altered when *p* < 0.05. Corresponding ion images of lipids that are found to be significantly altered in the LC-MS/MS data set. (C, D) Control vs ASO 15 days post administration: Volcano plot of identified lipids (LC-MS/MS data set) that classified with 3- and 4-stars in Lipostar. Positive and negative ionization mode measurements are combined. A lipid is significantly altered when *p* < 0.05. Corresponding ion images of lipids that are found to be significantly altered in the LC-MS/MS data set. Ion images are root-mean-square normalized.

## Conclusions

This study presents an enhanced multiomics (imaging) approach to study the distribution of an ASO that targets the mRNA that translates into the GABRG2 protein. Subsequently, the biological effect was studied, focusing on the metabolome, proteome, and lipidome. LC-MS/MS analysis was complemented with MALDI-MSI to perform pathway analysis and analyze the spatial distribution, respectively. The ASO’s PS-modified backbone was detected within kidney after 8 and 15 days post administration. However, the ASO fragment was detectable in brain tissue only 8 days post ASO administration. Next, the ASO’s effect on neurotransmitter distribution was analyzed. GABA expression was present in the whole brain but showed the highest level in the basal forebrain. By inhibition of GABRG2, a reduction trend in GABA was observed in most brain regions. Dopamine was mainly localized in the striatum but seemed not to be affected by targeting of GABRG2. By inhibiting the formation of GABRG2, we observed alterations in the activity, regulation, and plasticity of synapses (Figure 5). Glutamate receptors were also significantly altered in ASO-treated groups, suggesting tight regulation between GABA and glutamate. Since neurotransmitter receptors are membrane proteins, we also assessed the lipidome. Glycerophospholipids were mainly affected by the ASO, 8 and 15 days post administration. In conclusion, by using a multiomics (imaging) approach, we demonstrated that inhibiting GABRG2 by the use of an ASO affects neurotransmitters, proteome, and lipidome. The use of such a multiomics approach is highly valued in pharmaceutical research and development, since it integrates diverse molecular data sets to reveal novel insights into disease mechanism, drug response, and potential safety concerns, ultimately enhancing translational success rates.

**Figure 5:**
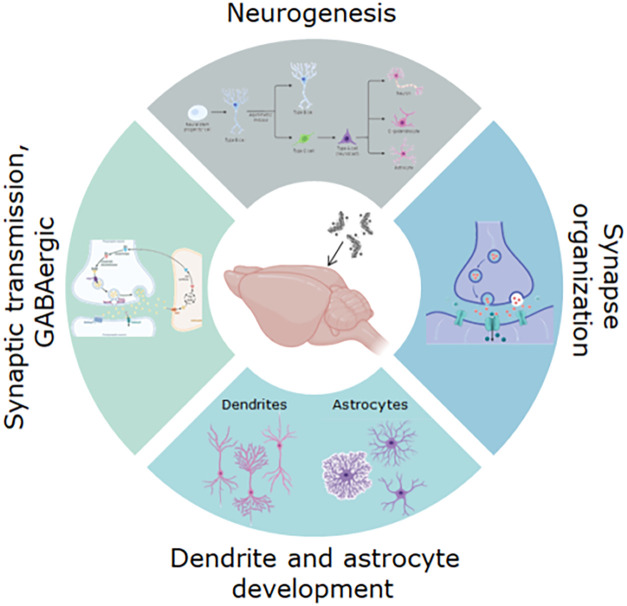
Concluding summary of the most important protein pathways that are affected as a consequence of ASO administration in brain tissue.

## Materials and Methods

### Chemicals and Reagents

Methanol (MeOH; ULC-MS grade), ethanol (EtOH; ULC-MS grade), HPLC-grade water, acetonitrile (ACN), dichloromethane (DCM), and chloroform (≥99%) were purchased from Fisher Scientific (Loughborough, Leicestershire, U.K.). Ammonium bicarbonate (ABC), dithiothreitol (DDT), eosin-Y (Avantor), formic acid (FA, ULC grade), Gill’s hematoxylin, iodoacetamide (IAM), norharmane, α-cyano-4-hydroxycinnamic acid (CHCA), trifluoroacetic acid (TFA, ULC grade), xylene, paraformaldehyde (PFA), phosphate-buffered saline (PBS 1×), and methyl-tert-butyl ether (MTBE) were purchased from Sigma-Aldrich (Zwijndrecht, The Netherlands). Entellan was purchased from Merck (Burlington, MA). RapiGest SF was purchased from Waters (Milford). Trypsin/LysC was obtained from Promega (Madison). FMP10 was purchased from Tag-ON (Uppsala, Sweden). The neurotransmitter standards serotonin (5-HT), γ-aminobutyric acid (GABA), dopamine hydrochloride (DA), and taurine were purchased from Merck (Burlington, MA). The isotope-labeled neurotransmitter standards GABA-2,2,3,3,4,4-d6 (GABA-d6) and DA-1,1,2,2-d4 hydrochloride (DA-d4) were purchased from Merck (Burlington, MA).

### Animal Experiments

The animals used in this study were handled and maintained in accordance with the general requirements of European Directive 2010/63/UE on the Protection of Animals Used for Scientific Purposes. Male Wistar rats (SPF Glx/BRL/Han) were intrathecally injected with the GABRG2 ASO. The ASO was diluted in artificial CerebroSpinal Fluid (aCSF), and the rats received a single dose of 1.2 mM. At the end of the experiment, rats were sacrificed, and brain and kidney samples were snap frozen for downstream analysis. Rats were sacrificed 8 and 15 days post ASO administration, collecting 3 control tissues and 2 dosed tissues per sacrifice.

### Tissue Sectioning Pathway Analysis

Fresh frozen brain and kidney tissues dosed with a vehicle (Ctrl) or GABRG2 ASO (sacrificed at day 8 and 15) were sectioned at 12 μm using a cryotome (Leica, Rijswijk, The Netherlands) at −20 °C and thaw-mounted onto idium-tin oxide (ITO, CG-40IN-S115, Delta Technologies) coated glass slides. Tissue slides were stored at −80 °C until further use.

### Visualization of the Phosphorothioate-Modified ASO Backbone via MALDI-MSI

Before matrix application, the slides were defrosted in a silica carrier box under a vacuum. Tissues were subsequently washed with DCM for 60 s and were dried in a desiccator. CHCA was used as a matrix and prepared by dissolving 10 mg/mL in 80% ACN and 1% TFA. CHCA was sprayed with an HTX M3+ sprayer (HTX Technologies, Chapel Hill, NC). The spraying parameters were as follows: temperature, 75 °C; nozzle velocity, 1200 mm/min; flow rate, 120 μL/min; number of passes, 4; track spacing, 1.5 mm; and nitrogen gas pressure, 10 psi. MALDI-MSI data was acquired on a timsTOF flex instrument (Bruker Daltonics GmbH, Germany) in negative ionization mode at a pixel size of 30 × 30 μm. The laser frequency was set to 10,000 Hz, and 300 shots were accumulated at each pixel. The TimsIn pressure was decreased to 1.8 mbar prior to acquisition. The method was externally calibrated using red phosphorus.

### On-Tissue Derivatization for Neurotransmitter Imaging

A deuterated neurotransmitter IS was prepared by dissolving GABA-d6, DA-d4, and 5-HT-d4 (0.01 mg/mL final concentration) in methanol. The deuterated neurotransmitter mixture was applied using the HTX M3+ sprayer (HTX Technologies, Chapel Hill, NC) using the following setting: temperature, 30 °C; nozzle velocity, 1200 mm/min; flow rate, 60 μL/min; number of passes, 16; track spacing, 2 mm; and nitrogen gas pressure, 10 psi. Samples were dried in a desiccator for 10 min prior to FMP10 reactive matrix application. FMP10 solution was prepared by dissolving 1.8 mg/mL in 70% ACN. FMP10 was sprayed with an HTX M3+ sprayer. Spraying parameters were as follows: temperature, 80 °C; nozzle velocity, 1100 mm/min; flow rate, 80 μL/min; number of passes, 20; track spacing, 2 mm; and nitrogen gas pressure, 6 psi. MALDI-MSI data was acquired on a timsTOF flex instrument (Bruker Daltonics GmbH, Germany) in positive ionization mode at a pixel size of 30 × 30 μm. The laser frequency was set to 1000 Hz, and 100 shots were accumulated at each pixel. The method was externally calibrated using red phosphorus and internally calibrated using the FMP10 cluster ion (*m*/*z* 555.2231) as lock mass.

A solariX FT-ICR mass spectrometer equipped with a 9.4T superconducting magnet (Bruker Daltonik GmbH, Bremen, Germany) was used to acquire high-mass-resolution imaging data of the neurotransmitters. The data was acquired in positive ionization mode at a pixel size of 50 × 50 μm in the mass range of 100–1000 *m*/*z*. The laser frequency was set to 2000 Hz, and 200 shots were accumulated at each pixel. The method was externally calibrated using red phosphorus and internally calibrated using the FMP10 cluster ion (*m*/*z* 555.2231) as lock mass.

### Spatial Lipidomics of ASO Dosed Brain Tissue

Brain sections containing slides were defrosted in a silica carrier box under vacuum. Consequently, the norharmane solution was prepared by dissolving 7 mg/mL in 33% MeOH and 66% chloroform. Norharmane was sprayed with the HTX M3+ sprayer using the following spraying parameters: temperature, 30 °C; nozzle velocity, 1200 mm/min; flow rate, 120 μL/min; number of passes, 15; track spacing, 3 mm; and nitrogen gas pressure, 10 psi. MALDI-MSI experiments were performed on a timsTOF flex instrument (Bruker Daltonics GmbH, Germany) in positive and negative ionization mode with a mass range of 300–1200 *m*/*z* at a pixel size of 30 × 30 μm. The laser frequency was set to 10,000 Hz, and 100 shots were accumulated at each pixel. The method was externally calibrated using red phosphorus and internally calibrated using PE 38:4, PI 38:4, and PI 36:4 (all [M – H]^−^) for negative ionization mode, as well as PC 32:0, PC 34:2, 36:4, and 38:4 (all [M + H]^+^) for positive ionization mode as the lock mass.

### Lipid Extraction of Whole Brain Tissue for LC-MS Analysis

Lipids were extracted using an MTBE extraction method, adapted from Matyash et al.^39^ In short, lipids were extracted from thinly sectioned (12 μm) brain tissue. Tissue sections were collected in 2.0 mL Eppendorf tubes that contained 375 μL of methanol and 1250 μL of MTBE that were vortexed for 10 s. Tissue suspensions were incubated for 1 h at RT and 500 rpm using a thermoshaker. Phase separation was introduced by the addition of 350 μL of Milli-Q water (to a final ratio of MTBE:MeOH:H_2_O of 10:2.5:3, v/v/v/) and centrifugation at 1000*g* for 10 min. The upper, organic phase, containing the lipids, was collected, and the lower phase was re-extracted with a solvent mixture with a composition equal to the upper phase (600 μL of MTBE, 180 μL of methanol, and 150 μL of Milli-Q water). After centrifugation, again, the upper phase was collected and combined with the previously obtained upper phase. The combined organic phases containing the lipids were dried in a vacuum centrifuge. The lipid fraction was reconstituted in IPA/ACN (50:50, v/v) and stored at −20 °C upon LC-MS/MS analysis.

### LC-MS/MS Lipidomics Analysis

Lipid analysis was performed on a Thermo Scientific (Dionex) Ultimate 3000 Rapid Separation UHPLC system with a Thermo Scientific Hypersil Gold C18 analytical column (10 cm, ID 2.1 mm, 1.9 μM). The UHPLC system was coupled to a high-mass-resolution Orbitrap MS Q-Exactive HF (Thermo Scientific) with a HESI-II source (Proxeon, Thermo Scientific). The spectrometer was programmed to run in data-dependent acquisition (DDA) mode and in positive and negative ionization modes. Here, MS1 data of *m*/*z* 200–1450 were acquired at a mass resolution of 60,000. In parallel, MS2 data was acquired in the ion trap with collision-induced dissociation (CID) using an isolation window of 1.7 Da and a mass resolution of 30,000. The lipid species were subsequently assigned using MS1 and MS2 spectra acquired from DDA measurements in Lipostar2 version 2.1.7. Lipid identifications for MALDI-MSI were assigned by linking MS1 precursor ions found in the MALDI-MSI measurements to the MS1 + MS2 *m*/*z* values found in the LC-MS/MS measurements using the LIPID MAPS database (3- and 4-star rating, Molecular Horizon, Bettona, PG, Italy). One-way ANOVA tests were performed to determine significance.

### On-Tissue Protein Digestion Followed by Peptide Imaging

Fresh frozen brain tissue was first washed and fixated by immersing the slides twice in ice-cold 100% EtOH for 2 min, once in 96% EtOH for 1 min, once in 70% EtOH for 1 min, and twice in ice-cold HPLC-grade water for 2 min. The slides were dried in a desiccator, followed by antigen retrieval using the Retriever 2100 (Aptum Biologics Ltd., Rownhams, U.K.) for 20 min at 121 °C. Citraconic anhydride buffer (pH 3.0) was prepared as described by Drake et al. The slide holder containing the slides was taken out of the antigen retriever and cooled in an ice bath for 5 min. Half of the buffer was then replaced with HPLC-grade water and placed back in an ice bath. This was repeated two more times, after which the slides were rinsed with HPLC-grade water and dried in the desiccator. The trypsin solution was freshly prepared by adding 200 μL of cold HPLC-grade water to 20 μg of trypsin. Trypsin was sprayed with an HTX M3+ sprayer (HTX Technologies, Chapel Hill, NC). Spraying parameters were as follows: temperature, 45 °C; nozzle velocity, 1200 mm/min; flow rate, 30 μL/min; number of passes, 8; track spacing, 2.5 mm; and nitrogen gas pressure, 10 psi. The slide was placed in an incubation chamber at 37 °C for 16 h. CHCA matrix solution (10 mg/mL in 70% ACN + 1% TFA) was applied with the HTX M3+ sprayer using the following parameters: temperature; 75 °C, nozzle velocity; 1200 mm/min, flow rate; 120 μL/min, number of passes; 4, track spacing; 1.5 mm, and nitrogen gas pressure of 10 psi. After being sprayed, slides were dipped in ice-cold 100 mM ammonium phosphate monobasic solution and dried vertically in a desiccator. MALDI-MSI data was acquired on a timsTOF flex instrument (Bruker Daltonics GmbH, Germany) in positive ionization mode at a pixel size of 30 × 30 μm. The laser frequency was set to 10,000 Hz, and 300 shots were accumulated at each pixel. The method was externally calibrated using red phosphorus before the imaging experiment and internally calibrated using Myelin Basic Protein (MBP) (*m*/*z* 1131.5674), actin (*m*/*z* 1198.7060), and MBP (*m*/*z* 1336.6314).

### MALDI-MSI Data Processing

Bruker Compass flexImaging 7.6 × 64 (Bruker Daltonik GmbH, Bremen, Germany) and SCiLS lab 2025b (SCiLS GmbH, Bremen, Germany) were used to process the acquired MALDI-MSI data. Data were RMS normalized or to the corresponding internal standard when applicable, and exported from SCiLS. Spectra were imported in mMass (version 5.5.0), where peaks were picked after baseline, smoothing, and deisotoping correction. One-way and two-way ANOVA tests were performed in GraphPad Prism (Version 10.5.0). Brain regions were selected for analysis by creating regions of interest. Lipid and peptide masses were matched and identified based on the results of the lipidomics and proteomics LC-MS/MS data sets.

### Protein Digestion for LC-MS/MS Proteomics Analysis

Whole tissue sections (12 μm thickness) were collected in a 1.5 mL Eppendorf that contained 50 mM ABC buffer. The samples were shortly centrifuged at 15,000*g* and were collected at the bottom of the tube. Next, 2.2 μL of 0.1% RapiGest was added to the sample and incubated for 10 min at RT, with shaking at 800 rpm. Subsequently, samples underwent reduction by the addition of DTT (200 mM in 50 mM ABC for final [DTT] = 10 mM), which was incubated for 40 min at 800 rpm and 56 °C. Next, samples underwent alkylation by the addition of IAM (400 mM in 50 mM ABC for final [IAM] = 20 mM), followed by incubation for 10 min at 800 rpm and RT. Lastly, DTT (final [DTT] = 10 mM) was added and incubated for 10 min at 800 rpm and RT. For protein digestion, trypsin (final v/v = 15 μg/mL) was added prior to overnight incubation for 16 h at 37 °C and 800 rpm. After incubation, trypsin (final v/v = 5 μg/mL) and ACN (final [ACN] = 80%) were added to the samples, followed by a 3 h incubation at 800 rpm and 37 °C. After incubation, TFA (final [TFA] = 0.5%) was added, and the sample was incubated for 45 min at 37 °C and 800 rpm. Finally, the samples were centrifuged at 15,000*g* for 15 min at 4 °C. The resulting supernatant was collected in a new Eppendorf tube and concentrated in a SpeedVac and resuspended in 2% ACN and 0.05% TFA in HPLC-grade water. Protein samples were stored at −20 °C until further use.

### LC-MS/MS Proteomics Analysis

Peptide separation was achieved on a Thermo Scientific (Dionex) Ultimate 3000 Rapid Separation UHPLC system with a Thermo Scientific Acclaim PepMap C18 analytical column (150 × 0.75 mm, 3 μm). Peptide samples were desalted on an online insulated C18 trapping column. After desalting, peptides were chromatographically separated on the analytical column with a 110 min gradient from 5% to 45% ACN/0.1% FA and a flow rate of 300 nL/min. The UHPLC system was coupled to a high-mass-resolution Orbitrap MS Q-Exactive HF (Thermo Scientific) with a nano electrospray Flex ion source (Proxeon, Thermo Scientific). The spectrometer was programmed to run in data-dependent acquisition (DDA) mode in positive ionization mode using the following parameters: MS1 scans between 250 and 1250 *m*/*z* at a resolution of 120,000, followed by MS2 scans of the top 15 most intense ions at a resolution of 15,000.

### Protein Identification and Analysis

Protein identification was performed in Proteome Discoverer software version 2.2 (Thermo Scientific) with the Uniprot protein database *Rattus norvegicus* (TaxID 10,116). The following settings were used for the protein database search: trypsin was used as the enzyme with a maximum of two missed cleavage sites and a minimum peptide length of six amino acids. The mass window for the precursor was set at 350–5000 Da. The mass tolerances of the precursor and fragment were 10 ppm and 0.02 Da, respectively. Acetylation on the N-terminus and methionine oxidation were used as dynamic modifications, and carbamidomethylation was used as static modification. A strict false discovery rate (FDR) of 0.01 was used to estimate the confidence in the identification. Accession numbers of the proteins were used to assess the protein-encoding gene names via the UniProtKB database. One-way ANOVA tests were performed to determine significance. Pathway enrichment analysis was performed on the significantly altered protein by using the STRING database (version 88) and Reactome software.

### Peptide Identification in the MALDI-MSI Data Set

Next, PCA was performed using the following parameters: No denoising or Pareto scaling and with 10 components. Discriminative analysis was executed by performing a receiver operating characteristic (ROC) using an area under the curve (AUC) threshold of 0.7 and 0.3 for significance. For linking the peptide mass to the corresponding protein ID, the criteria included a minimum of three peptide peaks to correspond to the protein. All selected peptide peaks must have the same spatial distribution and had a maximum mass error of 5 ppm.

### Histological Staining

Hematoxylin and eosin (H&E) staining was performed on the same sections used for MALDI-MSI experiments. The residual matrix was removed by submerging in 70% EtOH for 3 min followed by Milli-Q water for 3 min. Staining was performed in hematoxylin for 3 min, followed by rinsing under running tap water for 5 min, eosin for 30 s, running tap water for 3 min, EtOH for 1 min, and xylene for 30 s. Coverslips were mounted on tissue sections using Entellan mounting medium. Optical images were acquired at 20× magnification using the Aperio CS2 digital pathology slide scanner (Leica Biosystems, Wetzlar, Germany).

## Supplementary Material



## Notes

Schematic view of an ASO structure (Supporting Information Figure S1); wash solution optimization to detect the ASOs’ PS-modified backbone fragment (Supporting Information Figure S2); DCM wash solution optimization to enhance the ASOs’ PS-modified backbone fragment (Supporting Information Figure S3); neurotransmitter distribution in sagittal brain tissue using FMP10 as derivatization reagent (Supporting Information Figure S4); GABA and DA expression in ASO dosed brain sections 15 days post administration (Supporting Information Figure S5); relative intensities of neurotransmitters per brain region (Supporting Information Figure S6); reactome protein pathway analysis of ASO dosed brain tissue 8 days post administration (Supporting Information Figure S7); (Spatial) proteomic pathway analysis of brain tissue 15 days post ASO administration (Supporting Information Figure S8); lipid classes detected in brain tissue (Supporting Information Figure S9); high-mass-resolution measurement of neurotransmitters (Supporting Information Table S1); significantly altered proteins ASO dosed brain 8 days post administration (Supporting Information Table S2); significantly altered proteins ASO dosed brain 15 days post administration (Supporting Information Table S3); significantly altered lipids ASO dosed brain 8 days post administration (Supporting Information Table S4); and significantly altered lipids ASO dosed brain 15 days post administration (Supporting Information Table S5) (PDF)
