# Supplementary material for: Mass Spectrometry Imaging Reveals the Distribution of a GABRG2 Targeting Antisense Oligonucleotide and Its Functional Effect in Rat Brain
Source: ACS Chem Neurosci. 2025 Oct 1;16(20):4064–75. doi: 10.1021/acschemneuro.5c00524 (PMC12532189; doi:10.1021/acschemneuro.5c00524)
Supplement: Supplementary file 1 [file cn5c00524_si_001.pdf]

Mass spectrometry imaging reveals the distribution of a GABRG2 targeting antisense oligonucleotide and its' functional effect in rat brain

Laura van der Vloet [1], Ronny Mohren [1], Christophe Bouillod [2], Georges Da Violante [2], Emre M. Isin [2], Ron M.A. Heeren [1], Pierre Barbier Saint Hilaire [2], and Michiel Vandenbosch [1*]

[1] The Maastricht MultiModal Molecular Imaging (M4I) institute, Division of Imaging Mass Spectrometry (IMS), Maastricht University, 6229 ER Maastricht, The Netherlands
[2] Institut de Recherche et Développement SERVIER Paris-Saclay, 22 route 128, 91190 Gif-sur-Yvette, France

Author list:
Laura van der Vloet - *The Maastricht MultiModal Molecular Imaging (M4I) institute, Division of Imaging Mass Spectrometry (IMS), Maastricht University, 6229 ER Maastricht, The Netherlands*
laura.vandervloet@maastrichtuniversity.nl
https://orcid.org/0000-0003-2478-8675

Ronny Mohren - *The Maastricht MultiModal Molecular Imaging (M4I) institute, Division of Imaging Mass Spectrometry (IMS), Maastricht University, 6229 ER Maastricht, The Netherlands*
r.mohren@maastrichtuniversity.nl
https://orcid.org/0000-0001-6673-5542

Christophe Bouillod - *Institut de Recherche et Développement SERVIER Paris-Saclay, 22 route 128, 91190 Gif-sur-Yvette, France*
christophe.bouillod@servier.com

George Da Violante - *Institut de Recherche et Développement SERVIER Paris-Saclay, 22 route 128, 91190 Gif-sur-Yvette, France*
georges.da-violante@servier.com

Emre M, Isin - *Institut de Recherche et Développement SERVIER Paris-Saclay, 22 route 128, 91190 Gif-sur-Yvette, France*
emre.isin@servier.com
https://orcid.org/0000-0003-3402-7685

Ron M.A. Heeren - *The Maastricht MultiModal Molecular Imaging (M4I) institute, Division of Imaging Mass Spectrometry (IMS), Maastricht University, 6229 ER Maastricht, The Netherlands*
r.heeren@maastrichtuniversity.nl
https://orcid.org/0000-0002-6533-7179

Pierre Barbier Saint Hilaire - *Institut de Recherche et Développement SERVIER Paris-Saclay, 22 route 128, 91190 Gif-sur-Yvette, France*
pierre.barbier-saint-hilaire@servier.com
https://orcid.org/0000-0002-3365-5316

Michiel Vandenbosch - *The Maastricht MultiModal Molecular Imaging (M4I) institute, Division of Imaging Mass Spectrometry (IMS), Maastricht University, 6229 ER Maastricht, The Netherlands*
m.vandenbosch@maastrichtuniversity.nl
https://orcid.org/0000-0002-0427-416X

Table of content

Suppl. Figure 1. Schematic view of an ASO structure
Suppl. Figure 2. Wash solution optimization to detect the ASOs' PS modified backbone fragment
Suppl. Figure 3. DCM wash solution optimization to enhance the ASOs' PS modified backbone fragment
Suppl. Figure 4. Neurotransmitter distribution in sagittal brain tissue using FMP10 as derivatization reagent
Suppl. Figure 5. GABA and DA expression in ASO dosed brain sections 15 days post administration
Suppl. Figure 6. Relative intensities of neurotransmitters per brain region
Suppl. Figure 7. Reactome protein pathway analysis of ASO dosed brain tissue 8 days post administration
Suppl. Figure 8. (Spatial) proteomic pathway analysis of brain tissue 15 days post ASO administration
Suppl. Figure 9. Lipid classes detected in brain tissue

Suppl. Table 1. High mass resolution measurement of neurotransmitters
Suppl. Table 2. Significantly altered proteins ASO dosed brain 8 days post administration
Suppl. Table 3. Significantly altered proteins ASO dosed brain 15 days post administration
Suppl. Table 4. Significantly altered lipids ASO dosed brain 8 days post administration
Suppl. Table 5. Significantly altered lipids ASO dosed brain 15 days post administration

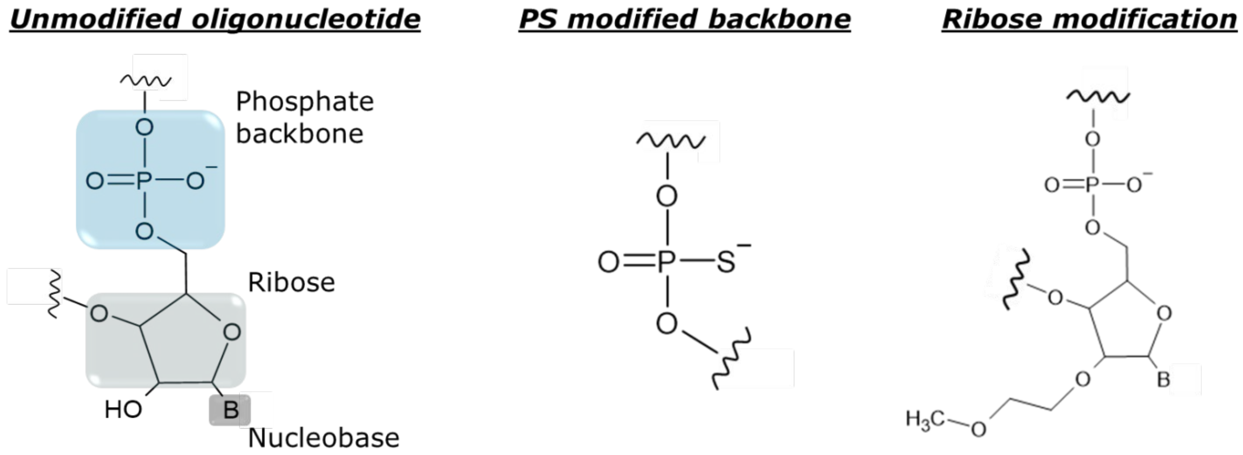

Suppl. Figure 1. Schematic view of an ASO structure. An unmodified ASO structure is presented. Mostly, an ASO contains 15-20 nucleobases containing multiple modifications, such as a modification on the phosphorothioate backbone and on the ribose.

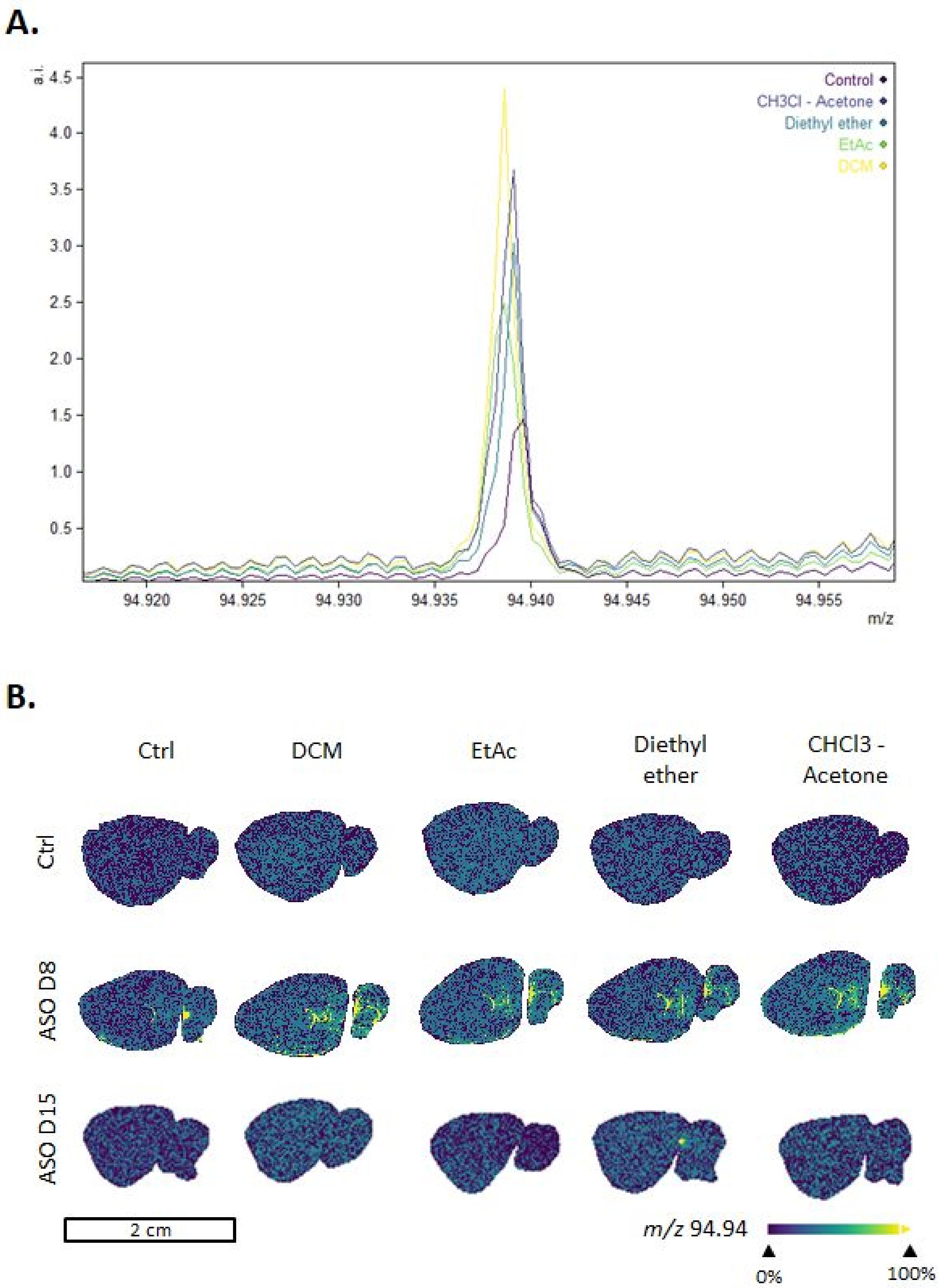

Suppl. Figure 2. Wash solution optimization to detect the ASOs' PS modified backbone fragment. A. Mass peak corresponding to the ASOs' PS modified backbone fragment (*m/z* 94.94) detected in brain tissue 8 days post ASO administration. Purple: control (no wash); Dark blue: Chloroform/Acetone; Blue: Diethyl ether; Green: Ethyl acetate; Yellow: Dichloromethane. **B.** Corresponding ion images of control, ASO D8, and ASO D15 brain tissues that underwent solvent washes for 1 min at room temperature prior MALDI-MSI analysis. Ion images are root mean square normalized.

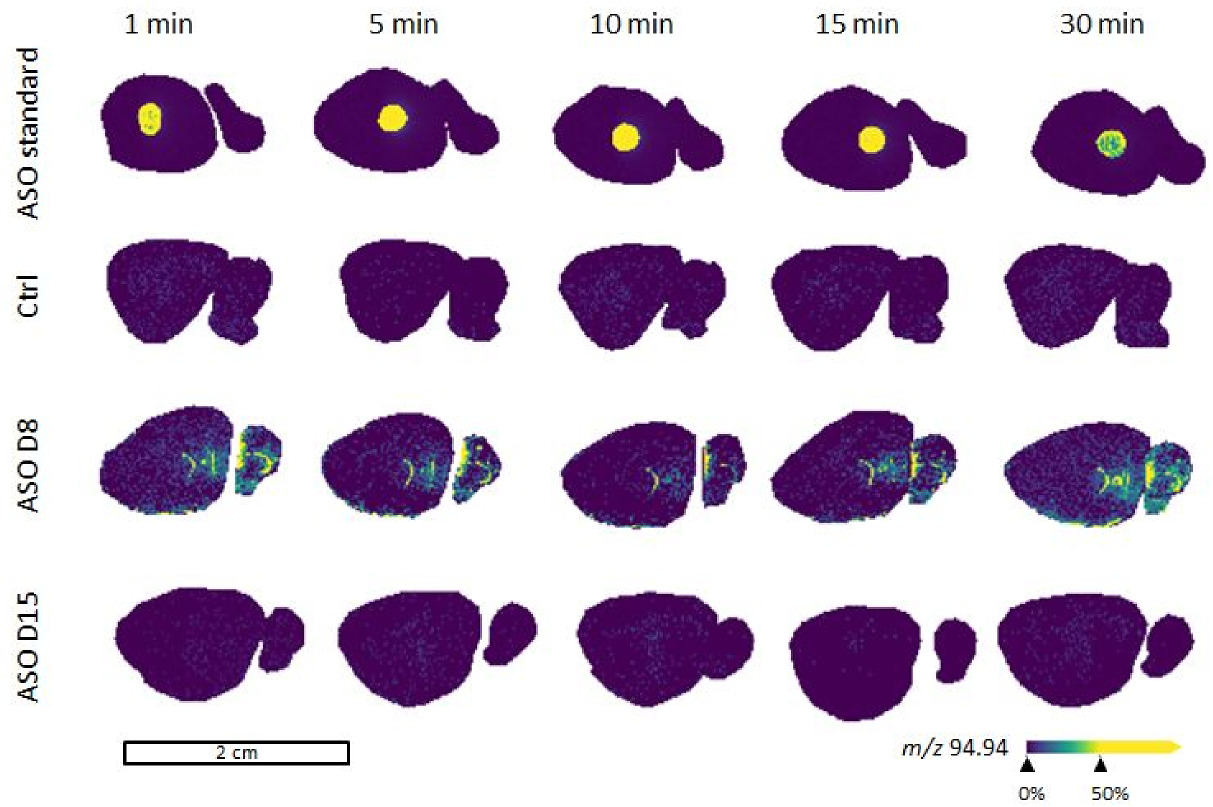

Suppl. Figure 3. DCM wash solution optimization to enhance the ASOs' PS modified backbone fragment detection. ASO standard (1 mg/mL) spotted on control brain tissue, control, ASO D8, and ASO D15 tissues were washed with dichloromethane prior MALDI-MSI analysis for different time points: 1 min, 5 min, 10 min, 15 min, and 30 min. The ASOs' PS modified backbone fragment was then visualized after root mean square normalization.

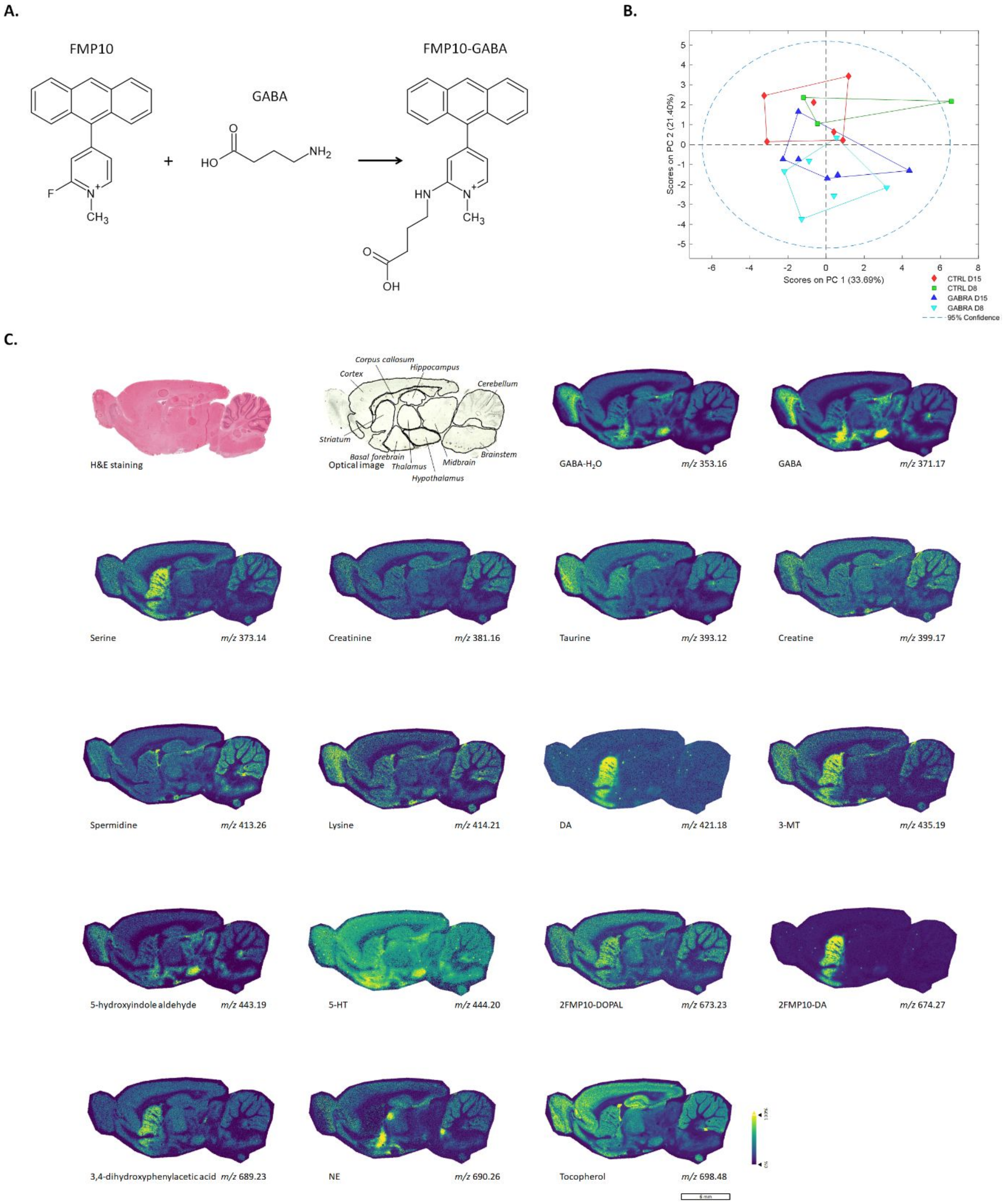

Suppl. Figure 4. Neurotransmitter distribution in sagittal brain tissue using FMP10 as derivatization reagent. A. Reaction between reactive matrix FMP10 and GABA to form the complex FMP10-GABA (*m/z* 371.17). **B.** PCA plot that was generated with a feature list containing all detected neurotransmitters utilizing MALDI-MSI analysis. **C.** Spatial distribution of neurotransmitters that are detected utilizing MALDO-MSI analysis. Ion images are presented after root mean square normalization. Optical image and corresponding H&E staining are presented with were used to annotate brain regions.

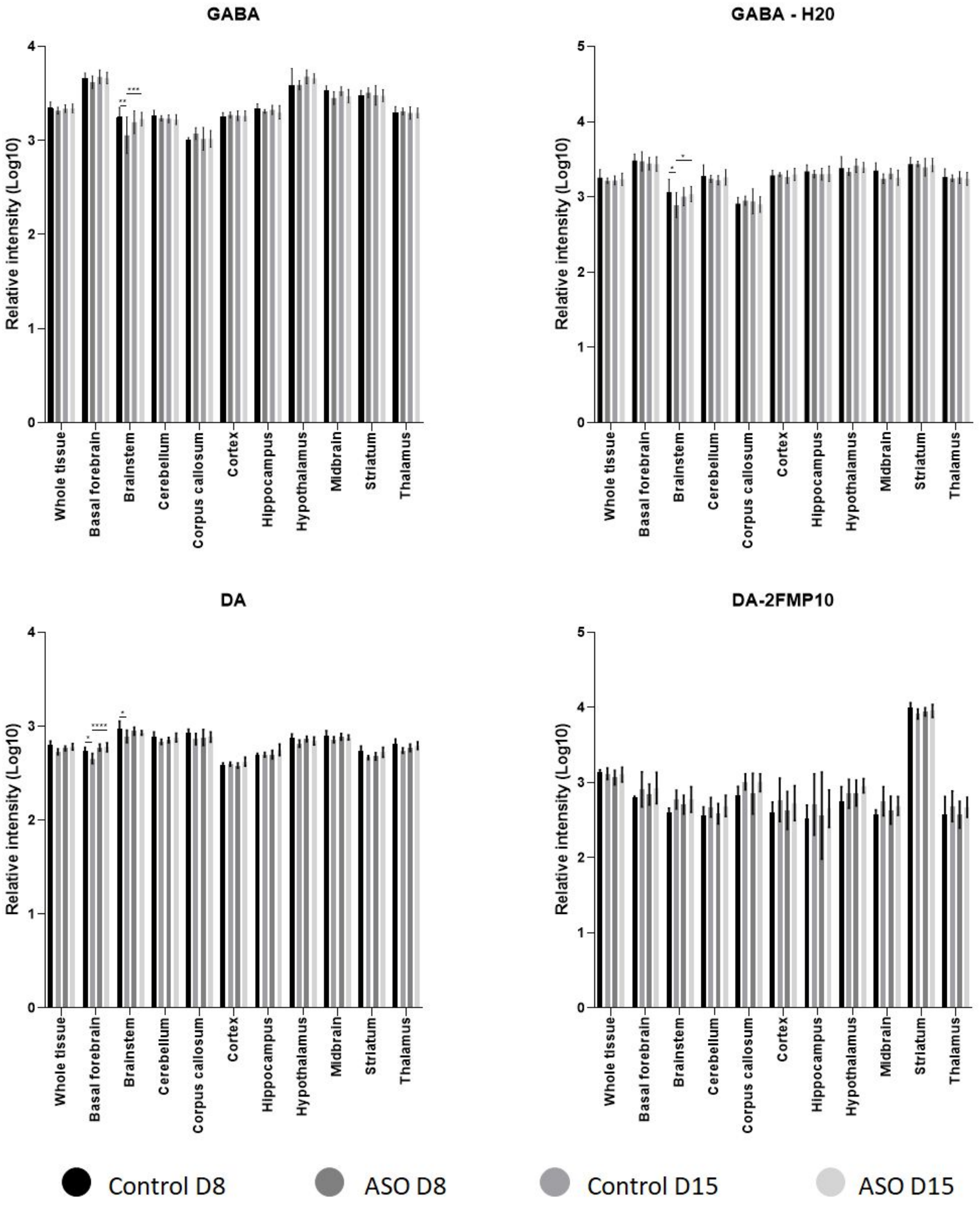

Suppl. Figure 5. Absolute intesntities of GABA and DA in ASO dosed brain sections 8- and 15- days post administration. Absolute intensities (Log10) are presented in bar plots of GABA and DA per brain region using MALDI-MSI. Data was normalized to their corresponding internal standard: GABA-d6 (m/z 377.21) and DA-d4 (m/z 425.21). Significance was determined by performing a 2-way ANOVA. Significance is presented as follows: *$p < 0.05$, **$p < 0.01$, ***$p < 0.001$, and ****$p < 0.0001$.

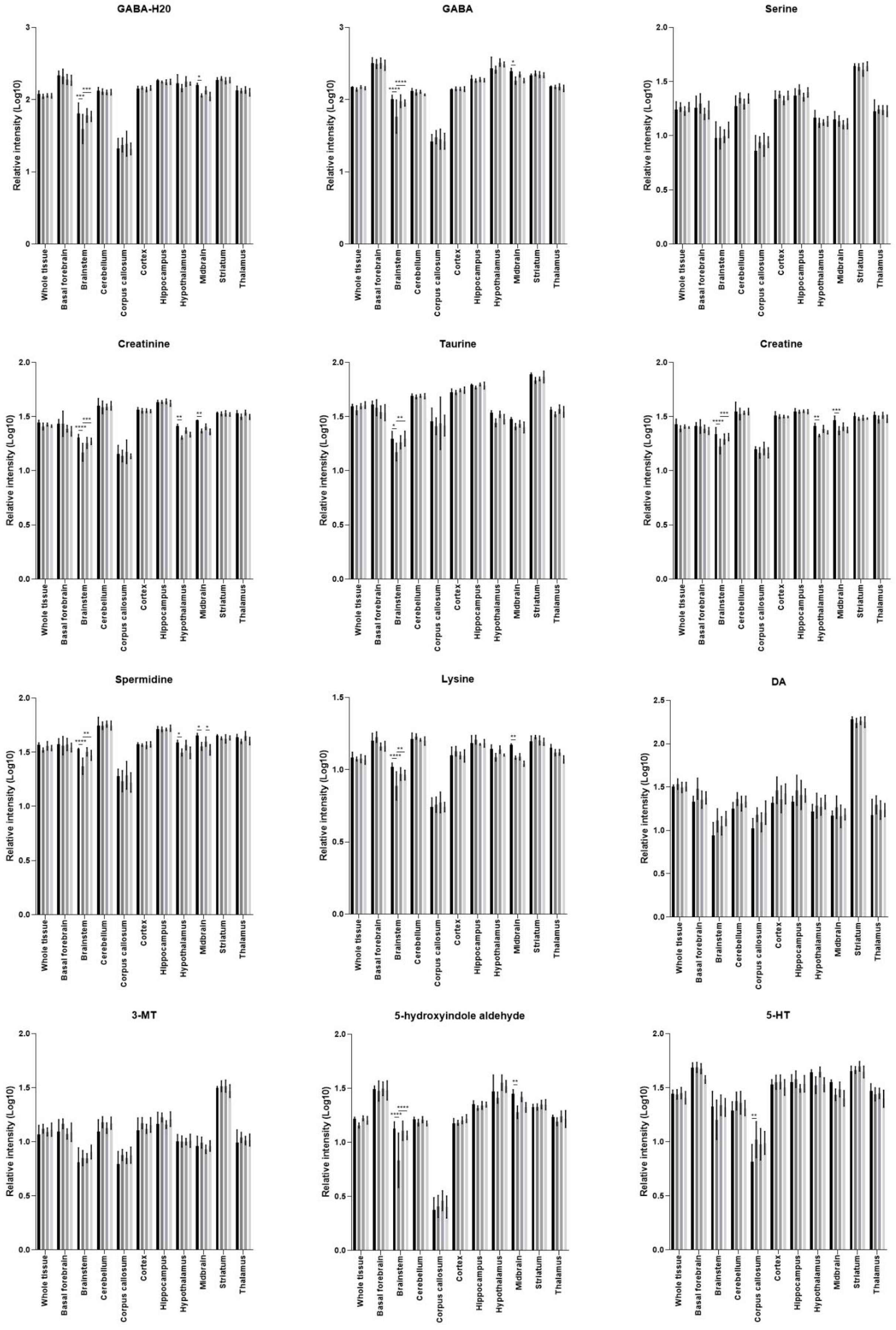
GABA-H20
GABA
Serine
Creatinine
Taurine
Creatine
Spermidine
Lysine
DA
3-MT
5-hydroxyindole aldehyde
5-HT
Relative intensity (Log10)
Whole tissue
Basal forebrain
Brainstem
Cerebellum
Corpus callosum
Cortex
Hippocampus
Hypothalamus
Midbrain
Striatum
Thalamus

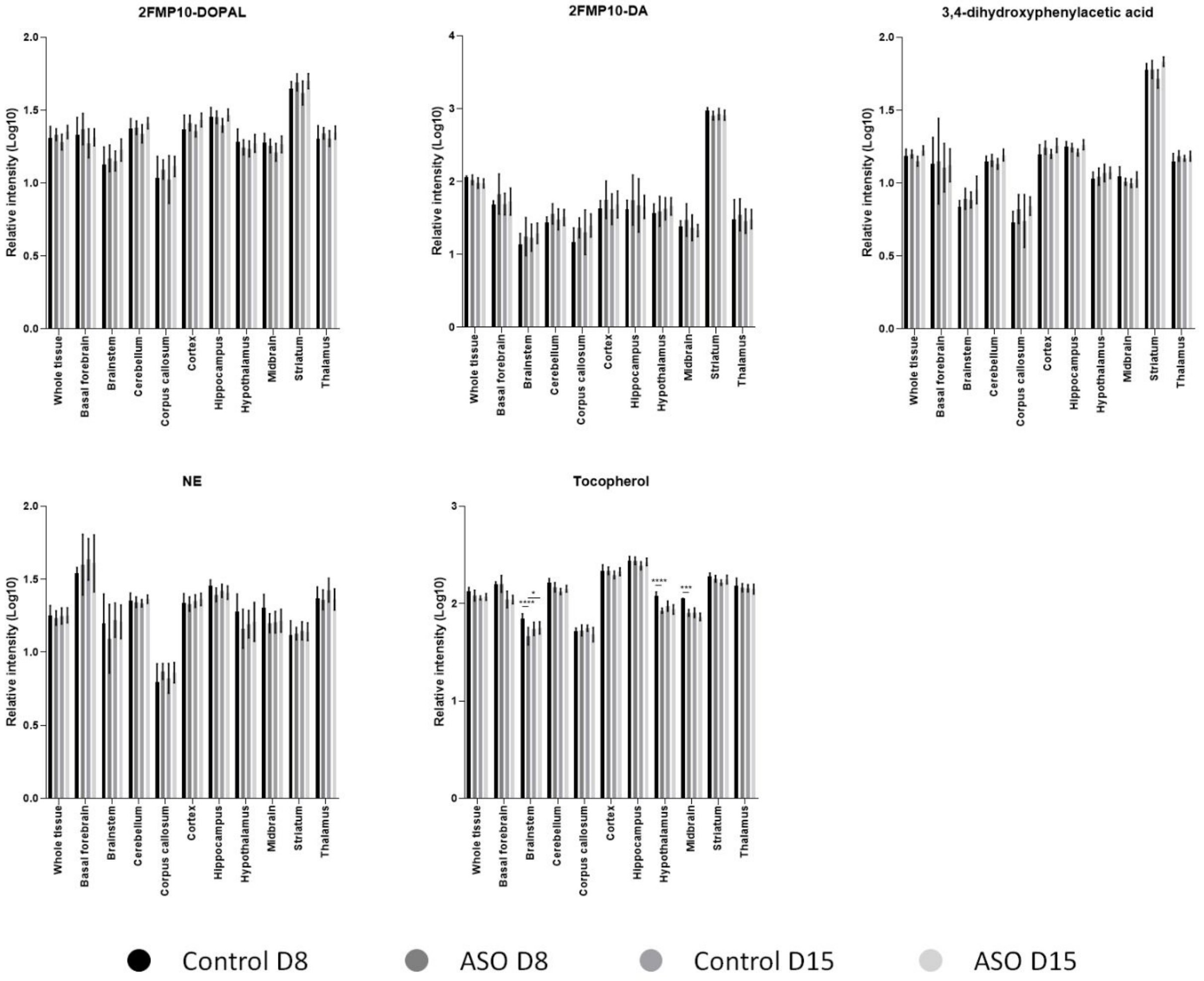

Suppl. Figure 6. Relative intensities of neurotransmitters per brain region. Relative intensities are presented in bar plots of each detected neurotransmitters per brain region using MALDI-MSI. Data was root mean square normalized, and significance was determined by performing a 2-way ANOVA. Significance is presented as follows: *p < 0.05, **p < 0.01, ***p < 0.001, and ****p < 0.0001.

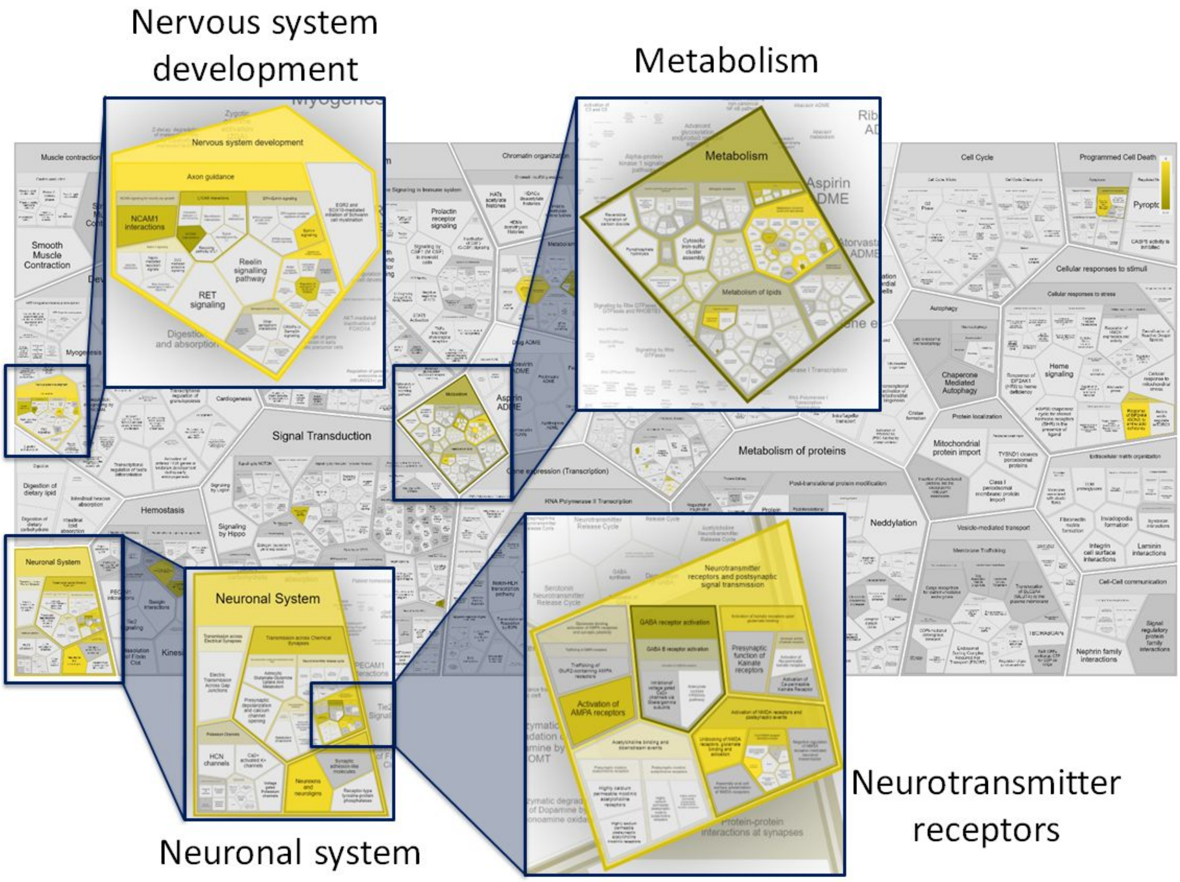

Suppl. Figure 7. Reactome protein pathway analysis of ASO dosed brain tissue 8 days post administration. Protein pathway analysis using the significantly altered proteins from the LC-MS/MS analysis. Proteins were considered to be significantly altered when $p < 0.05$ using a false discovery rate lower than 1%.

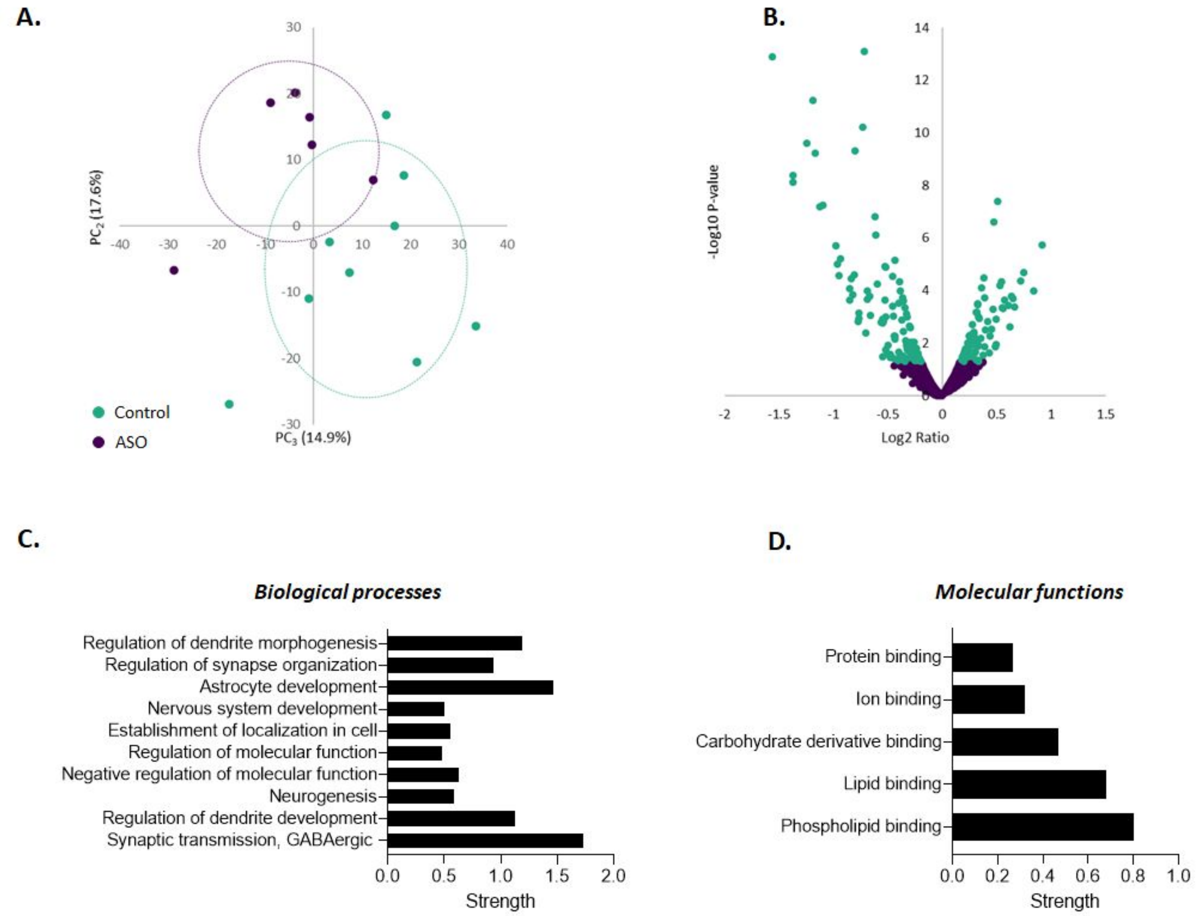

Suppl. Figure 8. (Spatial) proteomic pathway analysis of brain tissue 15 days post ASO administration. A. PCA analysis of identified proteins from the LC-MS/MS data set with a false discovery rate lower than 1%. In green, the control tissues are presented, and in purple, the ASO dosed (15 days after ASO administration) are presented. A 95% convenience ellipse is presented per experimental group **B.** A volcano of identified proteins from the LC-MS/MS data set with a false discovery rate lower than 1%. In green, the significantly ($p < 0.05$) altered proteins are presented. The left side of the volcano plot presents proteins that are upregulated in ASO dosed (15 days post administration) tissues, as where the right side presents proteins that are downregulated in ASO dosed tissues. **C. – D.** String protein pathways (top 10 GO terms related to biological processes and molecular functions) from significantly altered proteins in ASO dosed brain sections.

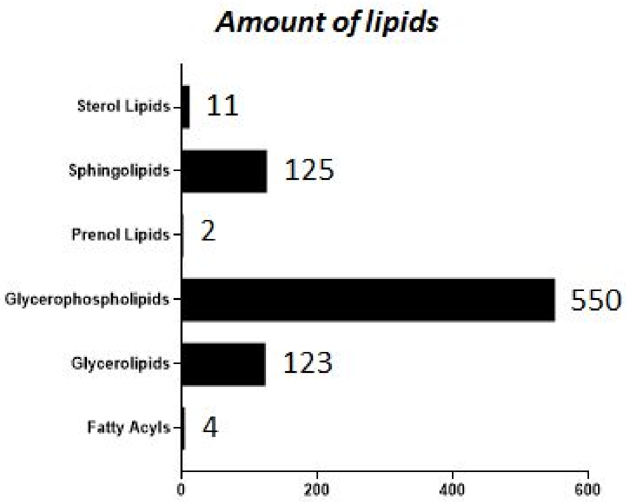

Suppl. Figure 9. Lipid classes detected in brain tissue. The total amount of lipids includes the lipids that were detected in positive and negative ionization mode by performing LC-MS/MS lipidomics analysis. Lipids were filtered on 3- and 4-stars convenience in Lipostar.

Suppl. Table 1. High mass resolution measurement of neurotransmitters.

m/z	Name
353.1646	GABA-H2O
371.1753	GABA
381.1709	Creatinine
393.1267	Taurine
421.1911	Dopamine
423.1815	Histidine
435.2068	3-MT
444.2071	5-HT
450.1700	HVA
673.2483	DOPAL-2FMP10
674.2798	Dopamine-2FMP10
689.2426	3,4-dihydroxyphenylacetic acid
690.2745	NE
698.4929	Tocopherol

Suppl. Table 2. Significantly altered proteins in ASO dosed brain 8 days post administration.

Protein ID	Description	GABRA D8	P-value
Q64303	Serine/threonine-protein kinase	↓	0.050
P29066	Beta-arrestin-1	↓	0.031
P31000	Vimentin	↓	0.026
P00564	Creatine kinase M-type	↓	0.022
Q4V8K5	BRO1 domain-containing protein	↓	0.022
O35094	Mitochondrial import inner membrane translocase subunit	↓	0.014

P57113	Maleylacetoacetate isomerase	↓	0.011
P11762	Galectin-1	↓	0.010
Q5PPG6	Nucleosome assembly protein 1-like 5	↓	0.009
P10818	Cytochrome c oxidase subunit 6A1, mitochondrial	↓	0.003
P02767	Transthyretin	↓	0.001
Q6LED0	Histone H3.1	↓	0.000
P13437	3-ketoacyl-CoA thiolase, mitochondrial	↓	0.000
P61314	Large ribosomal subunit protein eL15	↑	0.050
P13852	Major prion protein	↑	0.050
P56603	Secretory carrier-associated membrane protein 1	↑	0.050
Q9Z2X5	Homer protein homolog 3	↑	0.050
Q9Z272	ARF GTPase-activating protein	↑	0.045
Q6QIX3	Probable proton-coupled zinc antiporter	↑	0.045
P30009	Myristoylated alanine-rich C-kinase substrate	↑	0.043
Q8R431	Monoglyceride lipase	↑	0.043
P97710	Tyrosine-protein phosphatase non-receptor type substrate 1	↑	0.038
Q9QUH6	Ras/Rap GTPase-activating protein SynGAP	↑	0.038
P62813	Gamma-aminobutyric acid receptor subunit alpha-1	↑	0.036
P24054	SPARC-like protein 1	↑	0.036
P63159	High mobility group protein B1	↑	0.035
P63090	Pleiotrophin	↑	0.030
P97526	Neurofibromin	↑	0.029
P17074	Small ribosomal subunit protein eS19	↑	0.029
Q1M168	Caytaxin	↑	0.026
O35783	Calumenin	↑	0.021
P62246	Small ribosomal subunit protein uS8	↑	0.020
Q62936	Disks large homolog 3	↑	0.019
O08719	Ena/VASP-like protein	↑	0.018
P19491	Glutamate receptor 2	↑	0.014
Q63151	Fatty acid CoA ligase Acsl3	↑	0.014
P55053	Fatty acid-binding protein 5	↑	0.012
Q8CGU4	Arf-GAP with GTPase, ANK repeat and PH domain-containing protein 2	↑	0.011
A1L1K8	Intracellular hyaluronan-binding protein 4	↑	0.011
Q64620	Serine/threonine-protein phosphatase 6 catalytic subunit	↑	0.010
Q00960	Glutamate receptor ionotropic, NMDA 2B	↑	0.008
Q9JMB5	Proteasomal ubiquitin receptor ADRM1	↑	0.008
P43425	Guanine nucleotide-binding protein G(I)/G(S)/G(O) subunit gamma-7	↑	0.006
D4AEC2	Calmodulin-regulated spectrin-associated protein 2	↑	0.005
Q5XI72	Eukaryotic translation initiation factor 4H	↑	0.005
P38656	Lupus La protein homolog	↑	0.004
P09216	Protein kinase C epsilon type	↑	0.004
Q76EQ0	Serine racemase	↑	0.003
Q9JJW1	Tetraspanin-2	↑	0.002
Q5FVI4	Cell cycle exit and neuronal differentiation protein 1	↑	0.002
P54900	Sodium channel subunit beta-2	↑	0.002

O08875	Serine/threonine-protein kinase DCLK1	↑	0.002
Q62888	Neuroligin-2	↑	0.002
Q80ZA5	Sodium-driven chloride bicarbonate exchanger	↑	0.001
P55067	Neurocan core protein	↑	0.001
P62718	Large ribosomal subunit protein eL20	↑	0.001
Q63270	Cytoplasmic aconitate hydratase	↑	0.001
P32232	Cystathionine beta-synthase	↑	0.000
Q03344	ATPase inhibitor, mitochondrial	↑	0.000
O08873	MAP kinase-activating death domain protein	↑	0.000
P06302	Prothymosin alpha	↑	0.000
P35281	Ras-related protein Rab-10	↑	0.000
Q6J4I0	Protein phosphatase 1 regulatory subunit 1B	↑	0.000
Q05175	Brain acid soluble protein 1	↑	0.000
P11960	2-oxoisovalerate dehydrogenase subunit alpha, mitochondrial (Fragment)	↑	0.000
Q99P82	Claudin-11	↑	0.000
P07936	Neuromodulin	↑	0.000
Q9EPJ0	Nuclear ubiquitous casein and cyclin-dependent kinase substrate 1	↑	0.000
D3ZFB6	Proline-rich transmembrane protein 2	↑	0.000

Suppl. Table 3. Significantly altered proteins in ASO dosed brain 15 days post administration.

Protein ID	Description	GABRA D15	P-value
Q8K4G6	ADP-ribose glycohydrolase MACROD1 (Fragment)	↓	0.0479
Q9JJ50	Hepatocyte growth factor-regulated tyrosine kinase substrate	↓	0.0179
Q3B8Q2	Eukaryotic initiation factor 4A-III	↓	0.0145
P04639	Apolipoprotein A-I	↓	0.0073
P04218	OX-2 membrane glycoprotein	↓	0.0070
Q71UF4	Histone-binding protein RBBP7	↓	0.0064
Q5XIE0	Acidic leucine-rich nuclear phosphoprotein 32 family member E	↓	0.0062
Q498U4	SAP domain-containing ribonucleoprotein	↓	0.0053
Q80W83	Serine/threonine-protein phosphatase 2A 56 kDa regulatory subunit beta isoform	↓	0.0035
P43425	Guanine nucleotide-binding protein G(I)/G(S)/G(O) subunit gamma-7	↓	0.0032
Q9JJW1	Tetraspanin-2	↓	0.0032
P36972	Adenine phosphoribosyltransferase	↓	0.0012
P27274	CD59 glycoprotein	↓	0.0009
Q5HZY0	UBX domain-containing protein 4	↓	0.0009
Q5U316	Ras-related protein Rab-35	↓	0.0004
Q6AXQ0	SUMO-activating enzyme subunit 1	↓	0.0000
P14046	Alpha-1-inhibitor 3	↓	0.0000
P10960	Prosaposin	↓	0.0000
Q5XI72	Eukaryotic translation initiation factor 4H	↑	0.0484
P38656	Lupus La protein homolog	↑	0.0483
P97526	Neurofibromin	↑	0.0405

P09216	Protein kinase C epsilon type	↑	0.0184
P57113	Maleylacetoacetate isomerase	↑	0.0173
Q6AY65	Arfaptin-2	↑	0.0154
D4AEC2	Calmodulin-regulated spectrin-associated protein 2	↑	0.0142
Q62888	Neuroligin-2	↑	0.0128
O09175	Aminopeptidase B	↑	0.0123
O35764	Neuronal pentraxin receptor	↑	0.0120
Q8VHU4	Elongator complex protein 1	↑	0.0099
P13852	Major prion protein	↑	0.0057
Q9Z339	Glutathione S-transferase omega-1	↑	0.0045
Q63151	Fatty acid CoA ligase Acsl3	↑	0.0037
O70441	Synapsin-3	↑	0.0035
Q6P7S1	Acid ceramidase	↑	0.0034
P37361	Metallothionein-3	↑	0.0027
E9PSL7	Citron rho-interacting kinase	↑	0.0025
P11232	Thioredoxin	↑	0.0018
O54701	Sodium/potassium/calcium exchanger 2	↑	0.0018
Q07205	Eukaryotic translation initiation factor 5	↑	0.0015
P19804	Nucleoside diphosphate kinase B	↑	0.0010
Q63754	Beta-synuclein	↑	0.0009
Q9JKS6	Protein piccolo	↑	0.0006
P24054	SPARC-like protein 1	↑	0.0005
P06302	Prothymosin alpha	↑	0.0003
O08875	Serine/threonine-protein kinase DCLK1	↑	0.0003
B5DEH2	Erlin-2	↑	0.0002
P30009	Myristoylated alanine-rich C-kinase substrate	↑	0.0002
O08719	Ena/VASP-like protein	↑	0.0001
Q62829	Serine/threonine-protein kinase PAK 3	↑	0.0000
P55067	Neurocan core protein	↑	0.0000
Q63270	Cytoplasmic aconitate hydratase	↑	0.0000
A1L1K8	Intracellular hyaluronan-binding protein 4	↑	0.0000
P62845	Small ribosomal subunit protein uS19	↑	0.0000
Q03344	ATPase inhibitor, mitochondrial	↑	0.0000
P11960	2-oxoisovalerate dehydrogenase subunit alpha, mitochondrial (Fragment)	↑	0.0000
P63029	Translationally-controlled tumor protein	↑	0.0000
P62813	Gamma-aminobutyric acid receptor subunit alpha-1	↑	0.0000
O35142	Coatomer subunit beta'	↑	0.0000
P07633	Propionyl-CoA carboxylase beta chain, mitochondrial	↑	0.0000
Q5FVI4	Cell cycle exit and neuronal differentiation protein 1	↑	0.0000

Suppl. Table 4. Significantly altered lipids in ASO dosed brain 8 days post administration.

LC-MS/MS (m/z)	Theoretical value (m/z)	Lipid ID	Mass error (ppm)	Adduct	Polarity
808.5122	808.5123	PS 38:6	-0.12	[M+H]+	+

858.5252	858.5256	PS 40:6	-0.47	[M+Na]+	+
918.6864	918.6876	GalCer 38:1	-1.31	[M+H]+	+
794.5349	794.5341	PS P-38:4	1.01	[M-H]-	-
909.5453	909.5464	PI 38:4	-1.21	[M+Na]+	+
878.5934	878.5917	PC 40:6	1.93	[M+HCOO]-	-
806.5937	806.5917	PC 34:0	2.48	[M+HCOO]-	-
780.5888	780.5902	PE P-40:4	-1.79	[M+H]+	+
538.5188	538.5194	Cer 34:1	-1.11	[M+H]+	+
784.6657	784.6661	GalCer 40:1	-0.51	[M+H]+	+
776.5578	776.5589	PE P-40:6	-1.42	[M+H]+	+
634.5396	634.5405	DG 36:4	-1.42	[M+NH4]+	+
508.3395	508.3398	LPE 20:1	-0.59	[M+H]+	+
782.5685	782.5694	PC 36:4	-1.15	[M+H]+	+
686.5712	686.5718	DG 40:6	-0.87	[M+NH4]+	+
730.5735	730.5745	PE P-36:1	-1.37	[M+H]+	+

Suppl. Table 5. Significantly altered lipids in ASO dosed brain 15 days post administration.

LC-MS/MS (m/z)	Theoretical value (m/z)	Lipid ID	Mass error (ppm)	Adduct	Polarity
841.7167	841.7157	SM 44:2	1.19	[M+H]+	+
750.5307	750.5291	PC 30:0	2.13	[M+HCOO]-	-
702.5458	702.5443	PC O-32:1	2.14	[M-CH3]-	-
723.4981	723.497	PA 38:4	1.52	[M-H]-	-
704.5573	704.5589	PE O-34:1	-2.27	[M+H]+	+
724.5303	724.5287	PC O-34:4	2.21	[M-CH3]-	-
702.546	702.5443	PC P-32:0	2.42	[M-CH3]-	-
760.6198	760.6215	PE O-38:1	-2.24	[M+H]+	+
746.5362	746.5341	PS O-34:1	2.81	[M-H]-	-
480.3105	480.3096	LPE 18:0	1.87	[M-H]-	-
712.6458	712.645	TG 40:0	1.12	[M+NH4]+	+
860.6408	860.6386	PC 38:1	2.56	[M+HCOO]-	-
724.5258	724.5276	PE P-36:4	-2.48	[M+H]+	+
752.559	752.56	PE O-38:4	-1.33	[M-H]-	-
872.7094	872.7103	PC 42:1	-1.03	[M+H]+	+
728.5618	728.56	PE P-36:1	2.47	[M-H]-	-
922.5629	922.5604	PC 44:12	2.71	[M+HCOO]-	-
890.6877	890.6856	PC 40:0	2.36	[M+HCOO]-	-
502.2952	502.2939	LPE 20:3	2.59	[M-H]-	-
752.5611	752.56	PC O-36:4	1.46	[M-CH3]-	-
778.5621	778.5604	PC 32:0	2.18	[M+HCOO]-	-
805.6454	805.644	SM 38:0	1.74	[M+HCOO]-	-
872.6856	872.6832	GlcCer 42:1	2.75	[M+HCOO]-	-
812.5428	812.5436	PS 38:4	-0.98	[M+H]+	+

672.499	672.4974	PE P-32:1	2.38	[M-H]-	-
464.3154	464.3146	LPE O-18:1	1.72	[M-H]-	-
732.589	732.5902	PE O-36:1	-1.64	[M+H]+	+
716.5247	716.5236	PE 34:1	1.54	[M-H]-	-
760.6195	760.6215	PE O-38:1	-2.63	[M+H]+	+
718.5376	718.5392	PC 32:0	-2.23	[M-CH3]-	-
750.5459	750.5443	PE P-38:4	2.13	[M-H]-	-
810.5966	810.5983	PC 36:1	-2.10	[M+Na]+	+
728.5619	728.56	PC P-34:1	2.61	[M-CH3]-	-
502.2952	502.2939	LPE 20:3	2.59	[M-H]-	-
780.5681	780.5679	PC O-34:1	0.26	[M+Cl]-	-
1016.7281	1016.7255	GalCer 42:2	2.56	[M+HCOO]-	-
880.5127	880.5123	PS 44:12	0.45	[M+H]+	+
840.614	840.6124	PC O-38:4	1.90	[M+HCOO]-	-
859.6933	859.691	SM 42:1	2.68	[M+HCOO]-	-
730.5777	730.5756	PE P-36:0	2.87	[M-H]-	-
916.7038	916.7012	PC 42:1	2.84	[M+HCOO]-	-
744.5539	744.5549	PC 34:1	-1.34	[M-CH3]-	-
728.5621	728.56	PC P-34:1	2.88	[M-CH3]-	-
794.5349	794.5341	PS P-38:4	1.01	[M-H]-	-
744.5567	744.5549	PE 36:1	2.42	[M-H]-	-
790.632	790.632	PC 36:0	0.00	[M+H]+	+
